# Alpha-2 Adrenoreceptor Antagonist Yohimbine Potentiates Consolidation of Conditioned Fear

**DOI:** 10.1093/ijnp/pyac038

**Published:** 2022-06-24

**Authors:** Matthias F J Sperl, Christian Panitz, Nadine Skoluda, Urs M Nater, Diego A Pizzagalli, Christiane Hermann, Erik M Mueller

**Affiliations:** Department of Psychology, Personality Psychology and Assessment, University of Marburg, Marburg, Germany; Department of Psychology, Clinical Psychology and Psychotherapy, University of Giessen, Giessen, Germany; Department of Psychiatry, Harvard Medical School, & Center for Depression, Anxiety and Stress Research, McLean Hospital, Belmont, Massachusetts, USA; Department of Psychology, Personality Psychology and Assessment, University of Marburg, Marburg, Germany; Department of Psychology, Experimental Psychology and Methods, University of Leipzig, Leipzig, Germany; Center for the Study of Emotion and Attention, University of Florida, Gainesville, Florida, USA; Department of Clinical and Health Psychology, University of Vienna, Vienna, Austria; Department of Clinical and Health Psychology, University of Vienna, Vienna, Austria; Department of Psychiatry, Harvard Medical School, & Center for Depression, Anxiety and Stress Research, McLean Hospital, Belmont, Massachusetts, USA; Department of Psychology, Clinical Psychology and Psychotherapy, University of Giessen, Giessen, Germany; Department of Psychology, Personality Psychology and Assessment, University of Marburg, Marburg, Germany

**Keywords:** Fear conditioning, norepinephrine, dopamine, yohimbine, sulpiride

## Abstract

**Background:**

Hyperconsolidation of aversive associations and poor extinction learning have been hypothesized to be crucial in the acquisition of pathological fear. Previous animal and human research points to the potential role of the catecholaminergic system, particularly noradrenaline and dopamine, in acquiring emotional memories. Here, we investigated in a between-participants design with 3 groups whether the noradrenergic alpha-2 adrenoreceptor antagonist yohimbine and the dopaminergic D2-receptor antagonist sulpiride modulate long-term fear conditioning and extinction in humans.

**Methods:**

Fifty-five healthy male students were recruited. The final sample consisted of n = 51 participants who were explicitly aware of the contingencies between conditioned stimuli (CS) and unconditioned stimuli after fear acquisition. The participants were then randomly assigned to 1 of the 3 groups and received either yohimbine (10 mg, n = 17), sulpiride (200 mg, n = 16), or placebo (n = 18) between fear acquisition and extinction. Recall of conditioned (non-extinguished CS+ vs CS−) and extinguished fear (extinguished CS+ vs CS−) was assessed 1 day later, and a 64-channel electroencephalogram was recorded.

**Results:**

The yohimbine group showed increased salivary alpha-amylase activity, confirming a successful manipulation of central noradrenergic release. Elevated fear-conditioned bradycardia and larger differential amplitudes of the N170 and late positive potential components in the event-related brain potential indicated that yohimbine treatment (compared with a placebo and sulpiride) enhanced fear recall during day 2.

**Conclusions:**

These results suggest that yohimbine potentiates cardiac and central electrophysiological signatures of fear memory consolidation. They thereby elucidate the key role of noradrenaline in strengthening the consolidation of conditioned fear associations, which may be a key mechanism in the etiology of fear-related disorders.

**Significance Statement**

Hyperarousal (e.g., after traumatic events) leads to enhanced threat consolidation, which may play a crucial role in the etiology of pathological fear in posttraumatic stress and anxiety disorders. Rodent research has pointed to the important role of the noradrenergic system during hyperconsolidation of aversive associations. However, it is unclear whether noradrenergic arousal modulates neural markers of fear learning in humans. In the present study, we pharmacologically modulated central noradrenaline release after fear acquisition in a 2-day fear conditioning paradigm. We show that the alpha-2 adrenoreceptor antagonist yohimbine, given to participants directly after fear acquisition, leads to elevated electrocortical and cardiovascular threat responses 24 hours later. Heightened fear recall (for yohimbine) was indicated by potentiated amplitudes of the N170 and LPP event-related brain potentials (electroencephalography) and by elevated fear-conditioned bradycardia (electrocardiography). Our data suggest that yohimbine may provide a striking laboratory model to elucidate neural mechanisms in the etiology of clinical fear.

## Introduction

Heightened attention toward threat facilitates survival but can also contribute to clinical fear (Maddox et al., 2019). Whereas fear conditioning is construed as a core learning process in the etiology of anxiety and trauma-related disorders (Pittig et al., 2018), extinction learning is critical for the success of exposure therapy (Ressler, 2020). Noradrenergic (norepinephrine [NE]) activation, as induced by emotionally arousing experiences, is crucial for the formation and consolidation of new memory traces (Roozendaal et al., 2009; LaLumiere et al., 2017; Clewett and Murty, 2019). Exaggerated noradrenergic stimulation of the amygdala, hippocampus, and prefrontal brain areas plays a pivotal role in pathological fear, presumably mediated through aberrant conditioning and extinction (O’Donnell et al., 2004; Bowers and Ressler, 2015). Notably, overconsolidation of memories about life-threatening events due to amplified noradrenergic transmission may lead to intrusive memories (Nicholson et al., 2014), which are hard to extinguish (Miedl et al., 2020; Visser, 2020). Heightened threat responsiveness in posttraumatic stress disorder (PTSD) is mediated by hyperactivity of the locus coeruleus (Naegeli et al., 2018), the principal site for NE synthesis in the brain (Schwarz and Luo, 2015).

Rodent research has shown that stress-induced NE is critical for the consolidation of emotional memories (McGaugh, 2013; Bowers and Ressler, 2015). Optogenetic activation of locus coeruleus fibers leads to enhanced fear conditioning, presumably via NE release into the amygdala (Sears et al., 2013). The drug yohimbine acts as an antagonist at α2-autoreceptors in the locus coeruleus and stimulates NE release (Dunlop et al., 2012, 2015; Singewald et al., 2015). Of note, yohimbine facilitates fear consolidation (Gazarini et al., 2013) and generates a PTSD-like fear memory in rodents (Davis et al., 1979; Gazarini et al., 2015). In humans, yohimbine strengthens consolidation of fear-conditioned startle responses (Soeter and Kindt, 2011, 2012), in line with a hyperconsolidation hypothesis in PTSD (Nicholson et al., 2014). Yohimbine-induced stimulation of the NE system during initial fear consolidation may have long-lasting effects and lead to more stable memories about threat (Krenz et al., 2021).

In addition to its facilitating effect on fear consolidation, yohimbine may also enhance extinction (Cain et al., 2004; Hefner et al., 2008; Fitzgerald et al., 2014). This could have important clinical implications for the augmentation of exposure therapy (Mueller and Cahill, 2010). However, the results of rodent studies have been contradictory (Holmes and Quirk, 2010), and there is even evidence that yohimbine may enhance fear relapse (Morris and Bouton, 2007). Studies in humans suggest that yohimbine facilitates exposure therapy in PTSD (Tuerk et al., 2018), social anxiety disorder (Smits et al., 2014), and claustrophobia (Powers et al., 2009). However, others failed to replicate these effects for patients with a fear of flying (Meyerbroeker et al., 2012, 2018) and acrophobia (Meyerbroeker et al., 2018).

As outlined above, there is evidence that yohimbine facilitates fear consolidation. In contrast, some researchers have used yohimbine as a pharmacological complement to augment extinction learning during exposure therapy, but studies yielded mixed results (Holmes and Quirk, 2010). Experimental and therapeutic studies have either focused on fear consolidation or aimed at boosting extinction, but the 2 mechanisms have not been adequately differentiated. Here, we fill this gap by assessing yohimbine effects in an established paradigm (Mueller et al., 2014b) that allows us to distinguish the mechanisms specific to fear consolidation and extinction recall.

Furthermore, it remains unclear how yohimbine affects neural threat circuits in humans. Previous studies have tended to concentrate on peripheral measures (Soeter and Kindt, 2011, 2012; Tuerk et al., 2018; Esser et al., 2020; Kuehl et al., 2020); in the current study, we combined peripheral (skin conductance, heart rate) and central (electroencephalogram [EEG]) physiology to measure the effects of yohimbine. We were interested specifically in the N170 component and the late positive potential (LPP). The LPP is a reliable marker of conditioned fear (Panitz et al., 2015; Bacigalupo and Luck, 2018; Sperl et al., 2021), and the N170 has also been amplified when faces served as conditioned stimuli (CS) (Levita et al., 2015; Camfield et al., 2016; Sperl et al., 2021).

Besides its noradrenergic impact, yohimbine acts as an antagonist at dopaminergic D2-receptors (Scatton et al., 1980; Millan et al., 2000; Holmes and Quirk, 2010). In particular, yohimbine may block D2-autoreceptors and lead to elevated cortical dopamine (DA) levels (Gobert et al., 1997, 1998; Holmes and Quirk, 2010). So far, it has not been ascertained whether the effects of yohimbine can be ascribed to noradrenergic or dopaminergic signaling. As with noradrenergic pathways, the dopaminergic system plays a crucial role in acquiring emotional memories (Likhtik and Johansen, 2019; Papalini et al., 2020). To disentangle effects of yohimbine on NE and DA, we applied a between-participants design with 3 groups. In addition to the yohimbine and placebo groups, a third group received the DA D2-receptor antagonist sulpiride. We reasoned that, if yohimbine effects are driven by NE (vs DA) transmission, the pharmacological effects on fear conditioning and extinction should be specific to the yohimbine group and should not generalize to the sulpiride group.

In sum, animal and initial human studies suggest that yohimbine can boost fear consolidation, but neurophysiological mechanisms have rarely been studied in humans. As has been noted, there is also tentative evidence that yohimbine may facilitate fear extinction and thus enhance the efficacy of exposure therapy. Our study aims to elucidate (1) how yohimbine differentially affects fear consolidation and extinction learning, (2) which brain correlates underlie these mechanisms, and (3) whether the effects of yohimbine are driven specifically by noradrenergic stimulation.

## METHODS

### Participants

We recruited 55 healthy male students who were then randomly assigned to the 3 above-mentioned groups (exclusion criteria in Supplement A). One participant did not complete the study. Three participants were excluded because they fulfilled our criterion of “unlikely explicit contingency awareness” (i.e., higher awareness ratings for CS− than CS+ after acquisition, as defined by Sperl et al., 2019). Therefore, the final sample consisted of 51 participants (n = 17 yohimbine group, n = 16 sulpiride group, n = 18 placebo group). We tested males only because yohimbine’s neural effects are sex dependent (Schwabe et al., 2013) and estrogen levels modulate fear and extinction recall (Merz et al., 2018; Bierwirth et al., 2021). The study protocol was approved by the ethics committee of the German Psychological Society.

### Experimental Paradigm

**Figure 1.**
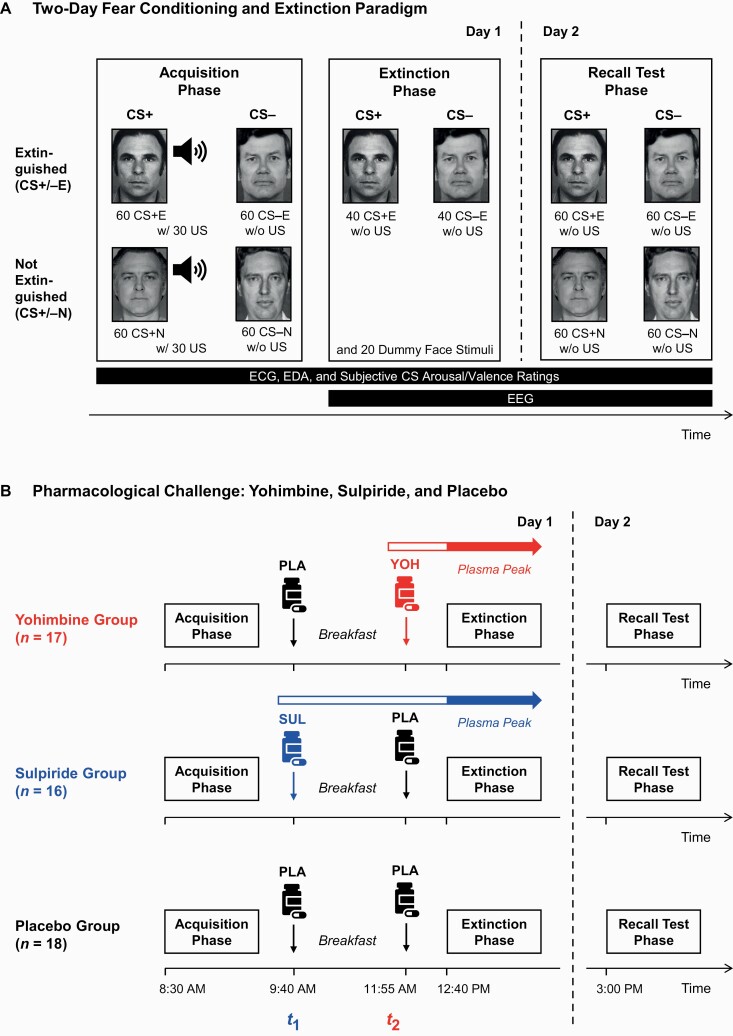
Experimental fear conditioning and extinction paradigm used in the present study. (A) Stimulus types and number of presentations during the 3 experimental phases. During acquisition training on the first day, 2 conditioned stimuli (2 CS+: “extinguished” [CS+E] and “non-extinguished” [CS+N]) were reinforced with (“w/”) an aversive unconditioned stimulus (US), which consisted of an unpleasant white noise burst (contingency of 50%). Conversely, 2 other conditioned stimuli (2 CS−: CS−E and CS−N) were not paired with the US (“w/o”). Afterward, participants underwent extinction training, during which only 1 CS+ (CS+E) and 1 CS− (CS−E) were shown. The CS+N and CS−N were not presented during extinction training. A novel face (“dummy stimulus”) was shown to maintain some variability of stimuli. On the second day, all stimuli were presented during a recall test without US presentation. To identify effects specific to fear vs extinction recall, we compared differential responses for non-extinguished stimuli (CS+N minus CS−N) with differential responses for extinguished stimuli (CS+E minus CS−E). Electrocardiogram (ECG) and electrodermal activity (EDA) were assessed during all stages. In addition to these peripheral measures, we recorded an electroencephalogram (EEG) during the day 1 extinction and day 2 recall stages. (B) Pharmacological challenge. Between fear acquisition and extinction stages, participants received an oral dose of either 10 mg of yohimbine hydrochloride (YOH, n = 17), 200 mg of sulpiride (SUL, n = 16), or a placebo pill (PLA, n = 18). All participants were tested at the same time of day to control for effects of circadian rhythms. Note that both substances (yohimbine and sulpiride) differ in the time they take to reach peak plasma concentration. Thus, sulpiride was administered at 9:40 am (= *t*_1_) and yohimbine at 11:55 am (= *t*_2_) to ensure that participants from both experimental groups reached peak plasma levels at a similar point. To guarantee successful blinding for experimenters and participants, each participant received 2 capsules. Participants in the sulpiride group received the active substance sulpiride 3 hours prior to extinction at *t*_1_ and a placebo pill at *t*_2_. Participants in the yohimbine group received yohimbine 45 minutes prior to extinction at *t*_2_ and a placebo pill at *t*_1_. For participants in the placebo group, both capsules contained placebo pills. All participants received a standardized light breakfast (water and 1–2 bread rolls with jam, hazelnut cocoa spread, cheese, or sausage) between the 2 capsules.

Participants underwent a well-established 2-day fear conditioning/extinction paradigm (Mueller et al., 2014b) with acquisition and extinction stages on day 1 and a recall test on day 2 (Figure 1A). During acquisition, 2 CS+ (CS+E [extinguished CS+] and CS+N [non-extinguished CS+]) and 2 CS− (CS−E [extinguished CS−] and CS−N [non-extinguished CS−]) were presented 60 times. Neutral faces (Ekman and Friesen, 1976) served as CSs (Supplement B). In differential fear conditioning paradigms, CS+ describes a CS that is paired with an aversive unconditioned stimulus (US). The CS− serves as a control stimulus that is never paired with the US. Both CS+ co-terminated with a white noise US (Sperl et al., 2016) at a partial reinforcement rate of 50%. Three hours after acquisition, participants began extinction training. One of the 2 CS+ (CS+E) and 1 of the 2 CS− (CS−E) were presented 40 times each in random order to extinguish threat responses to the CS+E. The other 2 CSs (CS+N and CS−N) and the US were not presented during extinction to leave learned responses to CS+N and CS−N fully intact. A novel face was shown 20 times to maintain some variability of stimuli.

Between acquisition and extinction, participants received (in a double-blind manner) an oral dose of either yohimbine hydrochloride (10 mg), sulpiride (200 mg), or a placebo. Yohimbine (45–75 minutes) and sulpiride (3–4 hours) vary in the time they take to reach peak plasma concentrations (Supplement C). To ensure peak plasma levels at a similar time prior to extinction, each participant ingested 2 capsules (Figure 1B). We assessed salivary α-amylase activity (sAA; Supplement D) to confirm yohimbine’s successful influence on central NE (Ehlert et al., 2006; Nater and Rohleder, 2009; Ditzen et al., 2014).

During a recall test approximately 26 hours after extinction, all stimuli (CS+E, CS+N, CS−E, CS−N) were presented 60 times each without any US presentation. By computing differential responses for extinguished (CS+/−E) and non-extinguished (CS+/−N) stimuli separately, extinction recall could be distinguished from fear recall on day 2. Participants were asked to rate each CS with regard to its associated arousal, valence, and perceived CS–US contingency (Supplement B).

### Physiological Data

Peripheral physiological data (skin conductance and electrocardiogram) were collected during all stages. Participants received yohimbine, sulpiride, or a placebo between acquisition and extinction. We were interested specifically in the pharmacological influences on neural threat signatures during subsequent extinction and fear/extinction recall 26 hours later. Hence, in addition to peripheral measures, we recorded EEG (64 channels) during the day 1 extinction and day 2 recall stages.

Recording and preprocessing details are described in Supplement E. Skin conductance response (SCR) scores (amplitude-sum within 1–5 seconds after CS onset) were calculated. To capture CS-evoked cardiac deceleration (Thigpen et al., 2017; Panitz et al., 2018), the mean heart period change from 2 to 5 seconds after CS onset was extracted. EEG data were high-pass (0.1 Hz) and notch-filtered (50 ± 2.5 Hz), corrected using independent component analysis (ocular artifacts), manually screened, and low-pass filtered (30 Hz). Afterward, we quantified N170 (145–185 milliseconds at left/right occipito-temporal electrodes T7/8, TP7/8, TP9/10, P7/8, PO9/10) and LPP (400–800 milliseconds at parieto-occipital electrodes P1, Pz, P2, PO3, POz, PO4, O1, Oz, O2) amplitudes (Supplement E).

### Statistical Analyses

Statistical tests were performed using SPSS 28 (IBM, Armonk, NY, USA), and *P* ≤ .05 (2-sided) was required to reach significance. Each experimental phase (day 1 acquisition, day 1 extinction, and day 2 recall test) was analyzed separately.

#### Affective CS Ratings and Peripheral Physiology

We expected higher ratings of arousal and negative valence after fear acquisition for both CS+ (CS+E, CS+N) compared with both CS− (CS−E, CS−N), which was assessed by contingency (CS+, CS−) × later extinction status (E = extinguished, N = not extinguished) × group (yohimbine, sulpiride, placebo) ANOVAs. At the peripheral physiological level, successful fear conditioning should be accompanied by higher SCRs (Mueller et al., 2014b) and relative cardiac deceleration (“fear-conditioned bradycardia”; Panitz et al., 2015) for both CS+ (CS+E, CS+N) compared with both CS− (CS−E, CS−N). For extinction, we computed contingency (CS+E, CS−E) × time (affective CS ratings: before/after extinction; skin conductance and heart period: first/last 10 trials) × group (yohimbine, sulpiride, placebo) ANOVAs because we expected a decrease of conditioned (CS+E vs CS−E) responses (Jentsch et al., 2020; Seligowski et al., 2020). At the beginning of the day 2 recall, contingency × extinction status × group ANOVAs were carried out. Successful fear and extinction recall on day 2 would be evident from larger affective and physiological responses for CS+N compared with CS−N, while responses following CS+E and CS−E should be similar. To achieve a sufficient signal-to-noise ratio for EEG recordings (Huffmeijer et al., 2014), we presented many CS trials during the day 2 recall stage (60 trials per CS type). Because of a rapid habituation of fear-conditioned SCRs (Sperl et al., 2019) and bradycardia (Panitz et al., 2018), peripheral measures of fear and extinction recall on day 2 were assessed during the first 10 trials.

#### Electroencephalography

As described above, we quantified N170 and LPP amplitudes, which are sensitive to the strength of conditioned threat (Camfield et al., 2016; Bacigalupo and Luck, 2018; Sperl et al., 2021). With regard to N170, an ANOVA including the within-participant factors contingency (CS+, CS−) × hemisphere (left, right) × electrode (T7/8, TP7/8, TP9/10, P7/8, PO9/10) and the between-participants factor group (yohimbine, sulpiride, placebo) was computed for day 1 extinction. To analyze LPP during extinction, we performed a contingency (CS+, CS−) × electrode (P1, Pz, P2, PO3, POz, PO4, O1, Oz, O2) × group (yohimbine, sulpiride, placebo) ANOVA. The N170 and LPP ANOVAs for day 2 fear/extinction recall included the additional within-participant factor extinction status (E, N).

Significant effects of mixed-model ANOVAs (including the between-participants factor group and several within-participant factors, as described above) were further analyzed using follow-up ANOVAs and *t* tests within groups. The Greenhouse-Geisser (1959) adjustment was used to correct for violations of sphericity.

### Data and Code Availability

Deidentified data along with a code-book and analysis scripts are posted at **https://doi.org/10.5281/zenodo.6833565.**

## RESULTS

### Manipulation Check Drug Administration: Salivary α-Amylase

**Figure 2.**
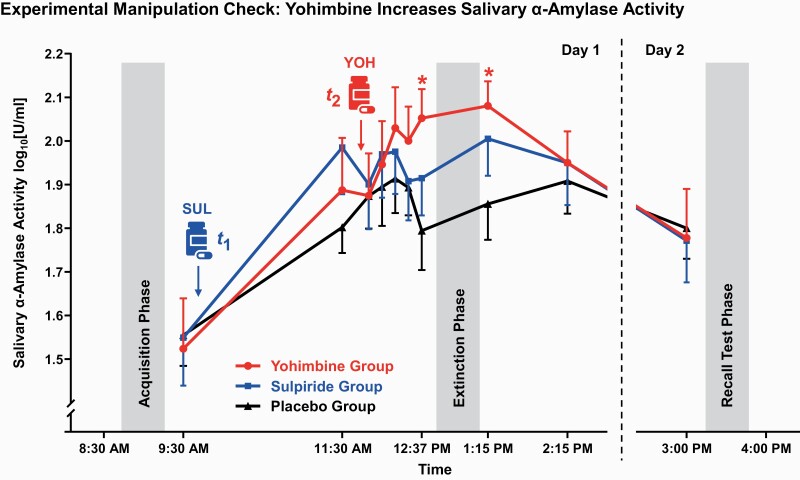
Between fear acquisition and extinction stages, participants received an oral dose of either 200 mg of sulpiride (SUL at *t*_1_; n = 16), 10 mg of yohimbine hydrochloride (YOH at *t*_2_; n = 17), or a placebo pill (n = 18). Salivary α-amylase activity (sAA) was assessed to confirm the successful influence of yohimbine on central noradrenaline (NE) release. Saliva samples were collected by using the passive drool method on both days at several time points (day 1: 9:30 am, 11:30 am, 11:57 am, 12:07 pm, 12:17 pm, 12:27 pm, 12:37 pm, 1:15 pm, and 2:15 pm; day 2: 3:00 pm). Compared with the placebo, yohimbine administration was associated with significantly elevated sAA activity directly before (12:37 pm) and after (1:15 pm) extinction training. Mean (± between-participants SEM) sAA activity values are displayed. All participants were tested at the same time of day to control for effects of circadian rhythms. *P ≤ .05.

Yohimbine administration (vs placebo) increased sAA activity (Figure 2) directly before (*t*_(32)_ = 2.34, *P* = .026) and after extinction (*t*_(32)_ = 2.26, *P* = .032), confirming the successful manipulation of NE release. There was no difference between groups before ingestion of the first capsule (*P* = .820) and before day 2 recall (*P* = .871). Sulpiride did not significantly elevate sAA activity (at all time points *P*s ≥ .147).

### Day 1 Fear Acquisition

Affective CS ratings and peripheral physiological responses confirmed successful fear conditioning (see Supplement G for details). The 2 CS+ (CS+E and CS+N), relative to the 2 CS− (CS−E and CS−N), evoked larger SCRs (contingency main effect, *F*_(1,48)_ = 15.87, *P* < .001) and stronger cardiac deceleration (“fear-conditioned bradycardia”; *F*_(1,47)_ = 44.94, *P* < .001) and were assessed as significantly more arousing (*F*_(1,48)_ = 27.36, *P* < .001) and unpleasant (*F*_(1,48)_ = 23.46, *P* < .001).

### Day 1 Fear Extinction

The contingency × time × group ANOVAs on CS arousal ratings and CS-evoked SCRs revealed significant contingency main effects. Specifically, the CS+E was still rated as significantly more arousing than CS−E (*F*_(1,48)_ = 20,89, *P* < .001) and generated elevated SCRs (*F*_(1,48)_ = 4.09, *P* = .049). ANOVAs on valence ratings, heart period, and N170/LPP did not yield significant effects involving contingency (*P*s ≥ .081).

During extinction, we did not observe significant interactions with the group factor (*P*s ≥ .081). This finding is in keeping with previous studies suggesting that yohimbine affects mainly consolidation processes (Soeter and Kindt, 2011, 2012), which occur predominantly during sleep (Pace-Schott et al., 2015); therefore, yohimbine effects would be expected especially on day 2.

### Day 2 Recall: Affective Ratings and Peripheral Physiological Data

The contingency × extinction status × group ANOVA for arousal ratings at the beginning of day 2 recall showed a significant contingency main effect (*F*_(1,48)_ = 25.74, *P* < .001). Both CS+E and CS+N were rated as significantly more arousing compared with CS−E and CS−N. Likewise, we observed elevated SCRs for both CS+ compared with both CS− (contingency main effect, *F*_(1,48)_ = 8.79, *P* = .005). The ANOVA on valence ratings did not yield any significant effects (*P*s ≥ .159). Contrary to our hypotheses, there were no significant interactions with the extinction status or group factors (*P*s ≥ .215) for affective ratings and SCRs.

**Figure 3.**
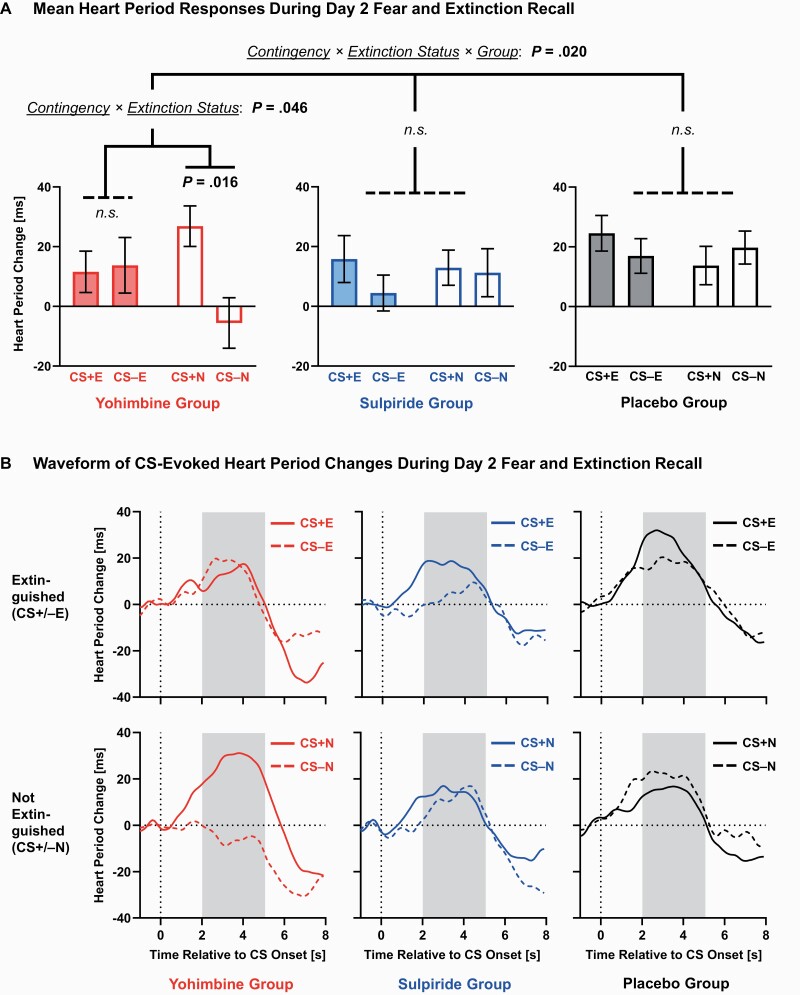
Fear-conditioned bradycardia (mean heart period change 2–5 seconds after the onset of conditioned stimuli [CS]) during day 2 recall. (A) The ANOVA for CS-evoked heart period changes revealed a significant contingency × extinction status × group interaction. Only the yohimbine group showed stronger cardiac deceleration for the non-extinguished CS+N compared with CS−N, indicating enhanced recall of fear-conditioned bradycardia. Mean (± within-participant SEM, adjusted within each group; O’Brien and Cousineau, 2014) heart period changes after CS onset are displayed. (B) The waveform of CS-evoked heart period changes is shown for extinguished (CS+E, CS−E; upper panels) and non-extinguished (CS+N, CS−N; lower panels) stimuli, separately for the yohimbine (n = 17; left panels), sulpiride (n = 16; middle panels), and placebo groups (n = 18; right panels). The time series of the interbeat interval was segmented into epochs ranging from –1 to 8 seconds relative to the CS onset, baseline corrected (1 second pre-CS), and averaged across trials for each CS type. Gray-shaded areas indicate time windows for statistical analyses.

The ANOVA on heart period data (Figure 3), however, revealed a significant contingency × extinction status × group interaction (*F*_(2,48)_ = 4.27, *P* = .020, $\eta_{p}^{2}$ =.151). To further assess the influence of the pharmacological manipulation on fear/extinction recall, we ran separate follow-up contingency × extinction status ANOVAs for each of the 3 groups. In contrast to prior studies (Panitz et al., 2015, 2018), we observed no significant main effects or interactions within the placebo (*P*s ≥ .261) and sulpiride (*P*s ≥ .370) groups; this indicates an absence of fear recall. Importantly, only the yohimbine group showed a significant contingency × extinction status interaction (*F*_(1,16)_ = 4.70, *P* = .046, $\eta_{p}^{2}$ = .227). For the yohimbine group, differential fear responses were significantly greater for non-extinguished vs extinguished stimuli. In particular, the non-extinguished CS+N was associated with stronger cardiac deceleration than the CS−N (*t*_(16)_ = 2.68, *P* = .016), reflecting successful fear recall. Conversely, there was no difference in the cardiac deceleration response between the extinguished CS+E and CS−E (*t*_(16)_ = −0.17, *P* = .870). In conclusion, yohimbine administration on day 1 was associated with enhanced recall of fear-conditioned bradycardia on day 2.

### Day 2 Recall: Electroencephalographic Data

#### N170

**Figure 4.**
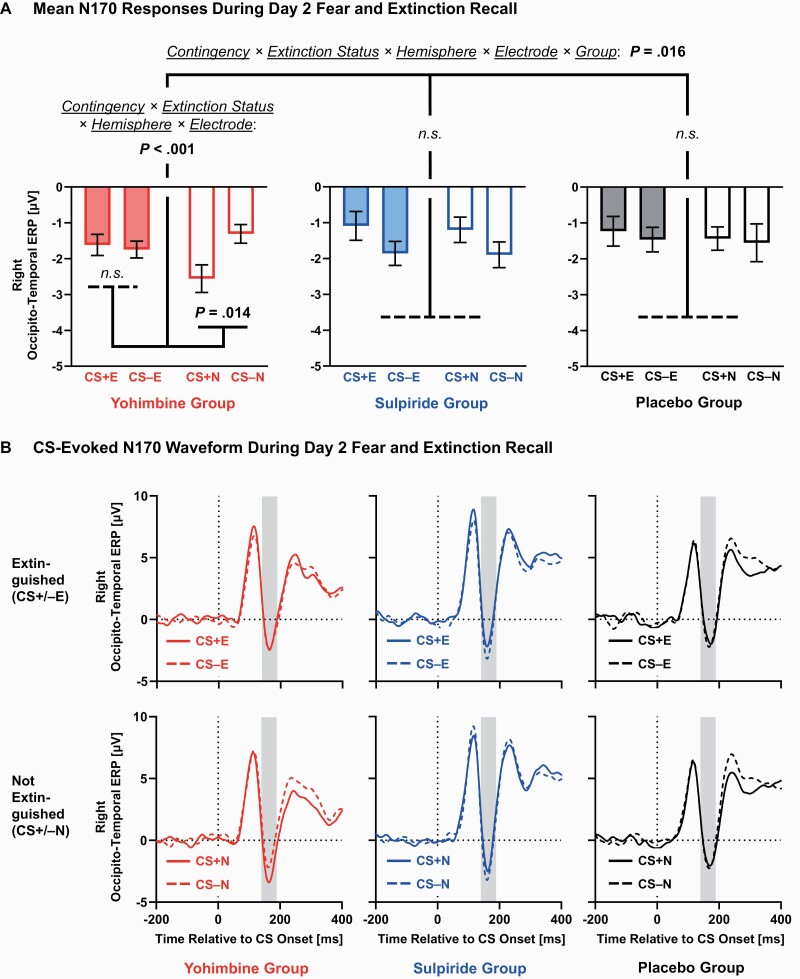
N170 component evoked by conditioned stimuli (CS) during day 2 recall. The ANOVA on mean amplitudes (145–185 milliseconds post-CS) yielded a significant contingency × extinction status × hemisphere × electrode × group interaction. Only the yohimbine group showed significantly larger (i.e., more negative) N170 amplitudes for the non-extinguished CS+N compared with CS−N, and effects were restricted to the electrodes TP10, P8, and PO10 over the right hemisphere. To illustrate (A) mean voltage changes (± within-participant SEM, adjusted within each group; O’Brien and Cousineau, 2014) and (B) event-related potential (ERP) waveforms, the electrode sites TP10, P8, and PO10 were averaged. The electroencephalographic data were referenced against electrode Cz, as this central reference highlights better the N170 at occipito-temporal electrodes (Joyce and Rossion, 2005). Gray-shaded areas indicate time windows for statistical analyses. The CS-evoked N170 waveform is shown for extinguished (CS+E, CS−E; upper panels) and non-extinguished (CS+N, CS−N; lower panels) stimuli, separately for the yohimbine (n = 17; left panels), sulpiride (n = 16; middle panels), and placebo groups (n = 18; right panels).

EEG responses closely mirrored the influence of yohimbine on fear-conditioned bradycardia. The ANOVA on N170 amplitudes (Figure 4) revealed a significant contingency × extinction status × hemisphere × electrode × group interaction (*F*_(8,192)_ = 2.60, *P* = .016, $\eta_{p}^{2}$_ _=_ _.098). Unexpectedly (but in line with our heart period data), follow-up contingency × extinction status × hemisphere × electrode ANOVAs for the placebo and sulpiride groups did not reach significance (with the exception of electrode main effects, *P*s ≤ .001). However, in the yohimbine group, we observed a significant contingency × extinction status × hemisphere × electrode interaction (*F*_(4,64)_ = 5.30, *P* < .001, $\eta_{p}^{2}$_ _=_ _.249). Convergent with prior observations that N170 responses are usually more pronounced in the right brain hemisphere (Eimer, 2011; Rossion and Jacques, 2012), significant contingency × extinction status interactions were confirmed at 3 right hemispheric electrodes: TP10 (*P* = .013), P8 (*P* = .006), and PO10 (*P* = .040). The N170 amplitude was significantly larger (more negative) for the CS+N compared with CS−N (TP10: *P* = .033; P8: *P* = .008; PO10: *P* = .020). In contrast, there was no difference between the CS+E and CS−E (TP10: *P* = .517; P8: *P* = .496; PO10: *P* = .774).

#### LPP

**Figure 5.**
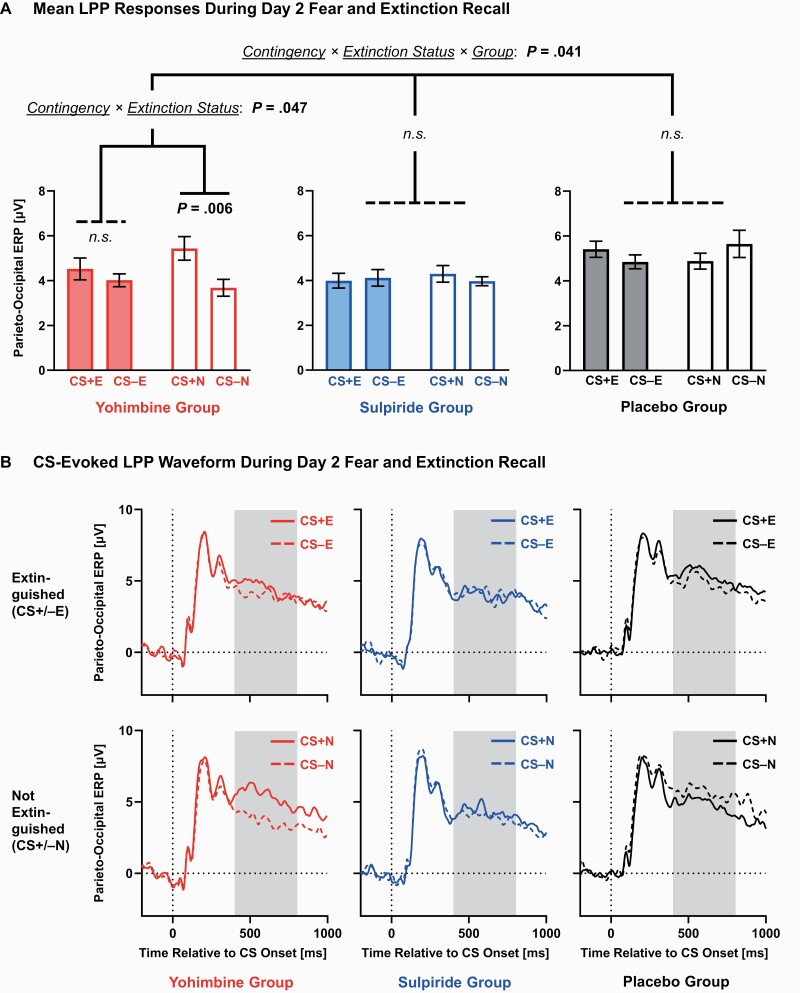
Late positive potential (LPP) component evoked by conditioned stimuli (CS) during day 2 recall. The ANOVA on mean amplitudes (400–800 milliseconds post-CS) yielded a significant contingency  ×  extinction status  ×  group interaction. Only the yohimbine group showed significantly larger (i.e., more positive) LPP amplitudes for the non-extinguished CS+N compared with CS−N. As there was no significant interaction with the electrode factor, all parieto-occipital electrodes (P1, Pz, P2, PO3, POz, PO4, O1, Oz, and O2) were averaged to illustrate (A) mean voltage changes (± within-participant SEM, adjusted within each group; O’Brien and Cousineau, 2014) and (B) event-related potential (ERP) waveforms. The electroencephalogram was referenced to the average of TP9 and TP10 (mastoids), which is consistent with the majority of LPP studies (Hajcak et al., 2012; Hajcak and Foti, 2020). The mastoid reference allows emotion-related LPP modulations to be better highlighted (Hajcak et al., 2012). Gray-shaded areas indicate time windows for statistical analyses. The CS-evoked LPP waveform is shown for extinguished (CS+E, CS−E; upper panels) and non-extinguished (CS+N, CS−N; lower panels) stimuli, separately for the yohimbine (n = 17; left panels), sulpiride (n = 16; middle panels), and placebo groups (n = 18; right panels).

For the LPP period (Figure 5), the ANOVA showed a significant contingency × extinction status × group interaction (*F*_(2,48)_ = 3.43, *P* = .041, $\eta_{p}^{2}$_ _=_ _.125). Follow-up ANOVAs for the placebo and sulpiride groups indicated significant electrode main effects (*P*s ≤ .024) but no further main effects or interactions (*P*s ≥ .198). Only the ANOVA for the yohimbine group revealed a significant contingency × extinction status interaction (*F*_(1,16)_ = 4.61, *P* = .047, $\eta_{p}^{2}$ = .224); this complemented our N170 results. We observed larger LPP amplitudes for CS+N compared with CS−N (*t*_(16)_ = 3.15, *P* = .006) within the yohimbine group. Conversely, there was no significant difference between LPP responses following CS+E and CS−E (*t*_(16)_ = 1.25, *P* = .229).

## Discussion

Noradrenergic hyperactivity plays a pivotal role in fear-related disorders (Krystal and Neumeister, 2009; LaLumiere et al., 2017; Giustino and Maren, 2018). Our primary goal was to elucidate NE effects on brain correlates of fear and extinction consolidation. Between conditioning and extinction, participants received either the α2-adrenoreceptor antagonist yohimbine (which leads to increased noradrenergic stimulation), the D2-receptor antagonist sulpiride (at low dose, which is thought to increase dopaminergic transmission), or a placebo. Sulpiride was added to exclude the possibility that yohimbine effects might be driven by DA because yohimbine (besides causing marked NE actions) also shows considerable affinity at D2-receptors (Scatton et al., 1980; Millan et al., 2000). The next day, we assessed peripheral and neural responses associated with fear and extinction recall. Notably, post-conditioning noradrenergic—but not dopaminergic—stimulation facilitated fear recall 1 day later, as manifested by fear-conditioned bradycardia and larger N170 and LPP amplitudes.

During day 2 recall, we compared differential responses to non-extinguished (CS+N minus CS−N) with extinguished (CS+E minus CS−E) stimuli to identify effects specific to fear vs extinction recall. Importantly, only participants who received yohimbine showed relative cardiac deceleration (bradycardia) for stimuli that had been fear conditioned and not extinguished (CS+N compared with CS−N). No effects for this contrast emerged for the placebo and sulpiride groups. Responses after extinguished CS+E were similar to CS−E in each of the 3 groups. Together, these results indicate that yohimbine selectively strengthened fear consolidation, resulting in robust fear recall on the second day.

Remarkably, neural responses during day 2 closely resembled the effects we observed on fear-conditioned bradycardia. Only participants in the yohimbine group showed significantly larger (more negative) amplitudes of the face-sensitive N170 component for the non-extinguished CS+N compared with CS−N, reflecting fear recall. This effect was absent in the sulpiride and placebo groups. The N170 component is a mid-latency, negative-going event-related potential component maximal over occipito-temporal scalp regions, which is particularly large in response to fear-conditioned (Pizzagalli et al., 2003; Dolan et al., 2006; Steinberg et al., 2012; Levita et al., 2015; Camfield et al., 2016; Mueller and Pizzagalli, 2016; Sperl et al., 2021) faces (Eimer, 2011; Schweinberger, 2011; Rossion and Jacques, 2012). Under the assumption that the N170 component is sensitive to variations in attention allocation (Eimer, 2000, 2018), elevated fear recall in the yohimbine group may thus indicate enhanced recruitment of attentional resources to faces that have been fear conditioned, consolidated under high levels of noradrenergic arousal, and not extinguished on the previous day. Interestingly, we observed larger N170 amplitudes for CS+N vs CS−N only at sensors over the right hemisphere, converging with the lateralization effects reported in previous fear-conditioning studies (Pizzagalli et al., 2003; Levita et al., 2015; but see Camfield et al., 2016). N170 amplitudes are typically larger over the right hemisphere (Eimer, 2011; Rossion and Jacques, 2012). This accords with the hypothesis of a right hemispheric advantage in face (Frässle et al., 2016) and danger-related emotion processing (Gainotti, 2019).

Like N170 effects, LPP amplitudes were enhanced for the CS+N vs CS−N, specifically in the yohimbine group. There was no significant difference between CS+N and CS−N in the sulpiride and placebo groups. The LPP is a late-latency parieto-occipital positivity (Hajcak et al., 2012, 2018), indicating sustained attention and elaborated neural processing (Wieser and Keil, 2020) due to stimulus significance (Hajcak and Foti, 2020). It is reliably elevated in response to fear-conditioned stimuli (Panitz et al., 2015; Bacigalupo and Luck, 2018; Seligowski et al., 2018; Stolz et al., 2019; Sperl et al., 2021) and is even sensitive to NE-related genetic influences on fear conditioning (Javanbakht and Poe, 2016; Panitz et al., 2018). LPP activity appears to be generated through the locus coeruleus NE system, which potentiates responding to arousing and motivationally significant stimuli (Nieuwenhuis et al., 2005; Hajcak et al., 2010; Hajcak and Foti, 2020). Collectively, our findings suggest that the administration of yohimbine strengthens neural signatures of conditioned fear that are linked to motivational NE circuits in the brain.

In contrast to some studies reporting threat responses with regard to N170 and LPP (Camfield et al., 2016; Bacigalupo and Luck, 2018; Sperl et al., 2021), we did not find N170/LPP threat modulations on day 2 in the placebo group. However, this observation is in line with previous studies that have applied very similar 2-day conditioning paradigms. In 2 prior datasets (Panitz et al., 2015; Muench et al., 2016), for example, we were unable to detect reliable conditioning effects on N170 or LPP amplitudes on the second day. In another study (Panitz et al., 2018), LPP amplitudes and fear-conditioned bradycardia on day 2 were elevated for CS+N compared with CS−N, but only in individuals of the Val/Val genotype of the *COMT* Val158Met polymorphism. Taken together, these findings suggest that robust threat responses can only be observed on day 2 after sufficient fear consolidation (e.g., as induced through NE release).

Regarding extinction recall, heart period, N170, and LPP responses did not differ between the CS+E and CS−E in any of the 3 groups. The lack of yohimbine effects on extinction learning adds to the considerable heterogeneity of findings from animal (Morris and Bouton, 2007; Holmes and Quirk, 2010) and human (Powers et al., 2009; Meyerbroeker et al., 2012, 2018; Smits et al., 2014; Tuerk et al., 2018) studies. While there is converging evidence that NE strengthens fear consolidation, it has been discussed that NE may have bidirectional (i.e., facilitating and inhibiting) effects on extinction (Giustino and Maren, 2018; Likhtik and Johansen, 2019; Giustino et al., 2020). Nevertheless, we may speculate as to why we did not observe yohimbine effects on extinction. Specifically, animal research suggests that yohimbine leads to faster fear extinction, that is, fewer trials are needed for successful fear reduction (Cain et al., 2004). We used a relatively high number of extinction trials to ensure a sufficient signal-to-noise ratio for the event-related potential computation (Huffmeijer et al., 2014). This may have resulted in a ceiling effect, so there may have been little left to be augmented by yohimbine (Meyerbroeker et al., 2018). Furthermore, in contrast with typical animal paradigms (Holmes and Quirk, 2010), acquisition and extinction took place on the same day. A longer interval between both experimental stages might be required to allow for sufficient fear memory consolidation before extinction (Maren, 2014; Dudai et al., 2015).

As discussed earlier, the pharmacology of yohimbine includes noradrenergic but also dopaminergic effects (Scatton et al., 1980; Millan et al., 2000; Holmes and Quirk, 2010). After yohimbine intake, sAA activity increased and was significantly larger relative to the placebo group, reflecting elevated release of central NE (Ehlert et al., 2006; Nater and Rohleder, 2009; Ditzen et al., 2014). To disentangle putative NE and DA effects of yohimbine, another group received the DA D2-receptor antagonist sulpiride. The absence of sulpiride effects, together with elevated sAA activity for the yohimbine group, suggests that yohimbine facilitated fear consolidation presumably through heightened NE release. By using sulpiride, we tried to mimic the effect of an increase in brain DA levels without the noradrenergic component of yohimbine. Nevertheless, we cannot exclude the possibility that concomitant facilitation of noradrenergic and dopaminergic release might be necessary to achieve the effect of yohimbine on fear consolidation. To rule out this alternative explanation, it would be necessary to include another experimental group, which receives a joint administration of yohimbine combined with a broad DA-receptor antagonist. In line with these interpretations, rodent studies showed that the combined DA and NE reuptake-blocker methylphenidate facilitates fear acquisition (Carmack et al., 2014b) and extinction (Abraham et al., 2012), but effects seem to depend on the chosen dose (Carmack et al., 2014a, 2014b). Haaker et al. (2013) demonstrated that administration of the DA precursor L-DOPA after fear extinction reduces the return of fear in both mice and humans. Together, these findings support the hypothesis that DA does indeed modulate fear learning, but dose and time of drug administration (e.g., before/after extinction) may be relevant. Sulpiride has been reported to facilitate extinction learning in mice (Ponnusamy et al., 2005), but another study has found attenuated fear extinction after sulpiride injection into the rat amygdala (Shi et al., 2017). These divergent findings (Ponnusamy et al., 2005; Yim et al., 2009; Dubrovina and Zinov’eva, 2010; Mueller et al., 2010; Stockhorst and Antov, 2015; Shi et al., 2017) may be explained by a recent study in rats suggesting that sulpiride can reduce fear expression but has no effect on acquisition/extinction learning (de Vita et al., 2021). Furthermore, depending on the chosen dose, sulpiride can lead to opposing effects due to pre- vs postsynaptic actions (Holmes and Quirk, 2010; Crockett and Fehr, 2014). In the present study, we used a relatively low dose of 200 mg, which is assumed to block primarily presynaptic autoreceptors, resulting in a net stimulatory effect on dopaminergic transmission (Tagliamonte et al., 1975; Mereu et al., 1983; Kuroki et al., 1999). Nevertheless, presynaptic and postsynaptic effects of sulpiride are not completely separable. In particular, it is not entirely clear where in the brain DA levels are increased by oral administration of low-dose sulpiride (Dodds et al., 2009; Ford, 2014; Brandão and Coimbra, 2019). Furthermore, the effects of sulpiride may vary between individuals depending on DA-related personality traits (Mueller et al., 2014a; Wacker and Smillie, 2015).

In addition to noradrenergic and dopaminergic actions, yohimbine also has significant affinity for serotonergic receptors (Millan et al., 2000), which has been largely ignored in the fear-conditioning literature (Holmes and Quirk, 2010). In the present study, we tried to control for dopaminergic mechanisms, but we cannot draw any conclusions about serotonergic actions of yohimbine. Several studies suggest that, in addition to NE and DA, modulations of the serotonergic system affect fear conditioning and extinction (Bauer, 2015). In light of the limited specificity of yohimbine, future studies should try to replicate our findings with a higher affinity and more selective α2-adrenoreceptor antagonist, such as atipamezole or MK-912 (Pettibone et al., 1989; Pertovaara et al., 2005; Proudman et al., 2022).

Hypervigilance is a core symptom of PTSD and other fear-related disorders (Javanbakht and Poe, 2016). It is characterized by abnormally elevated arousal and hyperactivity of the noradrenergic system (Morris et al., 2020). Yohimbine experimentally mimics the effects of noradrenergic arousal (Schwabe et al., 2013; LaLumiere et al., 2017). The NE system is highly vulnerable to sustained and uncontrollable stress, resulting in sensitization and persistent hyperarousal (Krystal and Neumeister, 2009; Kapfhammer, 2013). These processes lead to enhanced consolidation of emotional memories, which are more robust, detailed, vivid, and longer-lasting (Weymar and Hamm, 2013; McGaugh, 2013, 2015). Classical conditioning is an etiological mechanism, but not everybody who experiences traumatic events develops a mental disorder (Beckers et al., 2013; Duits et al., 2015; De Houwer, 2020). Notably, it has been suggested that high arousal levels after traumas play a key role in potentiated consolidation of CS–US associations, ultimately contributing to the development of pathological fear (Kapfhammer, 2013; Javanbakht and Poe, 2016). Specifically, higher heart rate shortly after a traumatic event has been reported in individuals who subsequently developed PTSD (Shalev et al., 1998; Bryant et al., 2000), which is consistent with overconsolidated memory networks due to heightened arousal (Javanbakht and Poe, 2016; Clewett and Murty, 2019; Krenz et al., 2021). Our data support this hypothesis; they demonstrate that noradrenergic hyperactivity after conditioning boosts fear consolidation. Translating this knowledge into clinical practice, this model would suggest that keeping arousal levels low in the aftermath of traumatic events might be a promising way to prevent later transition to PTSD or other fear-related psychopathology (Kapfhammer, 2013; Visser et al., 2015). Although our study proposes a notable model to stimulate innovative interventions for reducing pathological hyperconsolidation (Hoge et al., 2012; Astill Wright et al., 2019), clinical studies are needed to evaluate their efficacy.

To control for potential influences of gonadal hormone fluctuations on NE (Bangasser et al., 2016) and fear conditioning (Merz et al., 2018; Bierwirth et al., 2021), female participants were excluded. However, it is important to keep in mind that women are at twofold risk of developing PTSD and other fear-related disorders (Ramikie and Ressler, 2018; Christiansen and Berke, 2020); sex differences in the locus coeruleus NE system may explain elevated arousal levels in females (Bangasser et al., 2016). Further research is needed to clarify whether gonadal hormones modulate our findings.

EEG has limited spatial resolution. Its excellent temporal accuracy allowed us to capture yohimbine effects on brief neurophysiological processes during N170 and LPP periods, but little is known about brain circuits mediating noradrenergic actions in humans (Giustino and Maren, 2018). In rats, NE injection into the amygdala immediately after fear conditioning causes PTSD-like memory (Liu et al., 2019). Projections from the locus coeruleus might release NE into the amygdala (Likhtik and Johansen, 2019), or (vice versa) rapid amygdala processing may initiate locus coeruleus responses (Liddell et al., 2005). Although amygdala responses might explain threat-evoked potentiation of the N170 (Levita et al., 2015) and LPP (Bunford et al., 2018), electrophysiological methods have difficulties isolating neural signals from deep structures (Buzsáki et al., 2012; Keil et al., 2014; Jackson and Bolger, 2014). Future studies should combine our approach with functional magnetic resonance imaging to clarify the localization of underlying brain processes.

In conclusion, NE facilitates fear memory consolidation as quantified with cardiac deceleration and brain responses during the N170 and LPP time windows. Our results offer important neural evidence for yohimbine’s noradrenergic effects on fear consolidation in humans. Yohimbine provides a striking laboratory model to elucidate neural mechanisms in the etiology of clinical fear, which may open up promising paths for treatment.

## Supplementary Material

pyac038_suppl_Supplementary_Material

## Notes

The present study was supported by a grant from the Deutsche Forschungsgemeinschaft (DFG; German Research Foundation) to E.M.M. (grant no. DFG MU3535/2-1). M.F.J.S. received a Research Training Grant from the Society for Psychophysiological Research (SPR) for a 6-month research stay at McLean Hospital, Harvard Medical School (Belmont, MA, USA). Furthermore, M.F.J.S. was supported by the Research Training Group (RTG) 2271 “Breaking Expectations: Expectation Maintenance vs Change in the Context of Expectation Violations,” funded by the DFG (grant no. DFG 290878970-GRK2271, project 6). M.F.J.S. received a Poster Award for this project at the 57th Annual Meeting of the Society for Psychophysiological Research (SPR) in Vienna (Austria). M.F.J.S. and C.P. were also supported by the DFG grant MU3535/2-3, which was awarded to E.M.M. Over the past 3 years, D.A.P. has received consulting fees from Albright Stonebridge Group, Boehringer Ingelheim, Compass Pathways, Concert Pharmaceuticals, Engrail Therapeutics, Neumora Therapeutics (formerly BlackThorn Therapeutics), Neurocrine Biosciences, Neuroscience Software, Otsuka Pharmaceuticals, Sunovion Pharmaceuticals, and Takeda Pharmaceuticals; honoraria from the Psychonomic Society (for editorial work) and from Alkermes; and research funding from the National Institute of Mental Health (NIMH), the Dana Foundation, the Brain and Behavior Research Foundation, and Millennium Pharmaceuticals for activities unrelated to the current project. In addition, D.A.P. has received stock options from Compass Pathways, Engrail Therapeutics, Neumora Therapeutics, and Neuroscience Software. No funding from these entities was used to support the current work, and there are no conflicts of interest with the work conducted in this study. All views expressed are solely those of the authors. M.F.J.S., C.P., N.S., U.M.N., C.H., and E.M.M. reported no biomedical financial interests or potential conflicts of interest. M.F.J.S., C.P., C.H., and E.M.M. are members of the Center for Mind, Brain and Behavior (CMBB), a joint research center of the University of Marburg and the University of Giessen at the Research Campus Central Hessen. Open access publishing was supported by the Open Access Publication Fund of the University of Giessen.
