# Supplementary material for: Alpha-2 Adrenoreceptor Antagonist Yohimbine Potentiates Consolidation of Conditioned Fear
Source: Int J Neuropsychopharmacol. 2022 Jun 24;25(9):759–73. doi: 10.1093/ijnp/pyac038 (PMC9515133; doi:10.1093/ijnp/pyac038)
Supplement: pyac038_suppl_Supplementary_Material [file pyac038_suppl_supplementary_material.pdf]

Alpha-2 Adrenoreceptor Antagonist Yohimbine Potentiates Consolidation of Conditioned Fear

by

Matthias F. J. Sperl [1,2,3], **Christian Panitz** [1,4,5], **Nadine Skoluda** [6], **Urs M. Nater** [6], **Diego A. Pizzagalli** [3], **Christiane Hermann** [2], **Erik M. Mueller** [1]

[1] Department of Psychology, Personality Psychology and Assessment, University of Marburg, 35032 Marburg, Germany

[2] Department of Psychology, Clinical Psychology and Psychotherapy, University of Giessen, 35394 Giessen, Germany

[3] Department of Psychiatry, Harvard Medical School, & Center for Depression, Anxiety and Stress Research, McLean Hospital, Belmont, MA 02478, USA

[4] Department of Psychology, Experimental Psychology and Methods, University of Leipzig, 04109 Leipzig, Germany

[5] Center for the Study of Emotion and Attention, University of Florida, Gainesville, FL 32608, USA

[6] Department of Clinical and Health Psychology, University of Vienna, 1010 Vienna, Austria

Supplementary Material

Corresponding Author: Dr. Matthias F. J. Sperl

Current Address: Justus Liebig University Giessen, Department of Psychology,

Clinical Psychology and Psychotherapy, Otto-Behaghel-Str. 10F, 35394 Giessen, Germany;

matthias.sperl@psychol.uni-giessen.de; phone: +49 641 99 26086; fax: +49 641 99 26099

International Journal of Neuropsychopharmacology, https://doi.org/10.1093/ijnp/pyac038

ORCID IDs

Matthias F. J. Sperl: https://orcid.org/0000-0002-5011-0780

Christian Panitz: https://orcid.org/0000-0001-6692-8555

Nadine Skoluda: https://orcid.org/0000-0002-4140-2423

Urs M. Nater: https://orcid.org/0000-0002-2430-5090

Diego A. Pizzagalli: https://orcid.org/0000-0002-7772-1143

Christiane Hermann: https://orcid.org/0000-0001-5969-2898

Erik M. Mueller: https://orcid.org/0000-0002-8721-8963

Author Contributions

E.M.M. conceived the study design and acquired funding. M.F.J.S. and C.P. acquired the data. C.P. programmed the experiment and coordinated the data collection. M.F.J.S., C.P., N.S., and E.M.M. preprocessed and analyzed the data. N.S. and U.M.N. conducted biochemical sAA analyses. M.F.J.S. and E.M.M. drafted the manuscript, and C.P., N.S., U.M.N., D.A.P., and C.H. made critical revisions. M.F.J.S. created the figures. M.F.J.S. made the data, analysis scripts, and code-books publicly available at Zenodo. All of the authors interpreted and discussed the results, commented on the article, and approved the final manuscript for submission.

Data and Code Availability

De-identified data along with a code-book and analysis scripts are posted at https://doi.org/10.5281/zenodo.6833565.

A Exclusion Criteria for Participants

As described in the *Methods* section of the main text, we recruited 55 healthy male students at Justus Liebig University Giessen. One participant did not complete the study. Three subjects were excluded as they fulfilled our criterion of “unlikely explicit contingency awareness” (i.e., higher awareness ratings for CS- than CS+ after acquisition, as defined by Sperl et al., 2019; CS- = “conditioned stimulus *not* paired with the unconditioned stimulus”; CS+ = “conditioned stimulus paired with the aversive unconditioned stimulus“). Therefore, the final sample consisted of $N = 51$ participants ($n = 17$ yohimbine group, $n = 16$ sulpiride group, $n = 18$ placebo group).

We confirmed that participants were aware of the CS–US contingency (CS = “conditioned stimulus”; US = “unconditioned stimulus”) in each of the three groups (see Supplementary Figure S1). In the yohimbine group, contingency ratings were significantly higher for both CS+ compared with both CS- (CS+E versus CS-E: $t(16) = 11.96$, $P < .001$; CS+N versus CS-N: $t(16) = 14.98$, $P < .001$). Similarly, participants were contingency-aware in the sulpiride group (CS+E versus CS-E: $t(15) = 8.72$, $P < .001$; CS+N versus CS-N: $t(15) = 7.25$, $P < .001$). Finally, we verified that both CS+ (compared with both CS-) were associated with higher contingency ratings in the placebo group (CS+E versus CS-E: $t(17) = 11.25$, $P < .001$; CS+N versus CS-N: $t(17) = 11.90$, $P < .001$).

There were no significant group differences in age, body mass index (BMI), self-reported sleep quality/quantity measures (nights before day 1 and day 2), and trait/state anxiety (see Supplementary Table S1). All subjects were males, right-handed, and between the ages of 18 and 35 (mean age = 22.61 years, $SD = 3.05$ years). Exclusion criteria were (1) habitual use of tobacco, anorectics, or any illegal or prescription drugs; (2) BMI < 17 or > 30 kg/m^2; and (3) a history of neurological or cardiovascular diseases (e.g., hypertension or coronary heart disease), metabolic disorders, gastric or duodenal ulcers, gastrointestinal tract bleedings, hepatic or kidney diseases, or other chronic diseases that would require individual medical clarification. Participants underwent

a standardized clinical interview (Short Version of the Diagnostic Interview for Mental Disorders, Mini-DIPS; Margraf, 1994) to confirm the absence of mental disorders. In addition, participants were asked to refrain from alcoholic or caffeinated drinks, tea, juice, chewing gum, and strenuous exercise prior to the experiment (Bosch et al., 2011; Strahler et al., 2017). All subjects gave written informed consent to participate. They received monetary compensation (€10 per hour) or course credit.

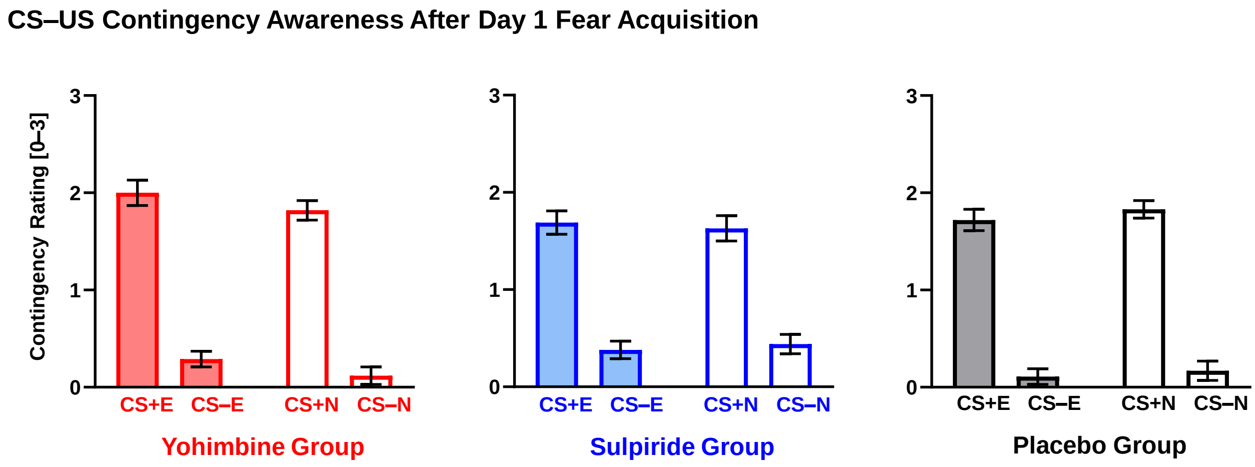

Supplementary Figure S1. Awareness of the CS–US contingency was confirmed in each of the three groups. After fear acquisition on day 1, participants were asked to indicate their subjective awareness of the CS–US contingency for each CS type (0 = “CS was never followed by US”; 3 = “CS was always followed by US”). Mean (± within-participant *SEM*, adjusted within each group; O'Brien and Cousineau, 2014) contingency ratings for each CS type are displayed.

Supplementary Table S1. Sample Characteristics: Age, Body Mass Index (BMI), Sleep, and Trait/State Anxiety Measures (Mean ± Standard Deviation). There were no significant differences between the three experimental groups.

Variable	Yohimbine Group<br>*n* = 17<br>*M* (± *SD*)	Sulpiride Group<br>*n* = 16<br>*M* (± *SD*)	Placebo Group<br>*n* = 18<br>*M* (± *SD*)	Between-Groups Statistical Comparison
Age and Body Mass Index (BMI)				
Age [Inclusion Criterion: 18–35 years]	22.24 (± 2.75)	22.63 (± 3.24)	22.94 (± 3.28)	$F(2,48) = 0.23$, $P = .796$
Actual Age Range: 18–32 years	19–28	18–29	19–32	
BMI [Inclusion Criterion: 17–30 kg/m²]	23.65 (± 3.13)	24.22 (± 3.20)	23.86 (± 2.22)	$F(2,48) = 0.17$, $P = .846$
Actual BMI Range: 17.73–29.94 kg/m²	19.25–29.32	17.73–29.73	20.99–29.94	
Sleep Measures[1]				
Sleep Quality Before Day 1 [1–5]	3.76 (± 0.75)	3.69 (± 0.60)	3.89 (± 0.58)	$F(2,48) = 0.42$, $P = .661$
Sleep Quality Before Day 2 [1–5]	4.29 (± 0.69)	4.50 (± 0.73)	4.11 (± 0.76)	$F(2,48) = 1.22$, $P = .306$
Hours Slept Before Day 1	6.47 (± 1.10)	6.70 (± 1.20)	6.53 (± 1.09)	$F(2,48) = 0.18$, $P = .837$
Hours Slept Before Day 2	7.71 (± 0.94)	8.28 (± 1.06)	7.78 (± 1.32)	$F(2,48) = 1.28$, $P = .288$
Tiredness Day 1 [1–4]	1.53 (± 0.51)	1.25 (± 0.45)	1.50 (± 0.51)	$F(2,48) = 1.58$, $P = .216$
Tiredness Day 2 [1–4]	1.12 (± 0.33)	1.06 (± 0.25)	1.11 (± 0.32)	$F(2,48) = 0.16$, $P = .853$
Trait and State Anxiety[2]				
STAI Trait Anxiety [20–80]	37.41 (± 9.19)	35.69 (± 5.49)	33.50 (± 5.76)	$F(2,48) = 1.37$, $P = .265$
STAI State Anxiety Day 1 [20–80]	31.53 (± 3.96)	33.37 (± 3.22)	34.83 (± 6.21)	$F(2,48) = 2.16$, $P = .126$
STAI State Anxiety Day 2 [20–80]	29.29 (± 3.57)	30.62 (± 4.94)	32.78 (± 7.98)	$F(2,48) = 1.58$, $P = .217$

[1]Sleep quality and quantity for the preceding night were assessed on both days at the beginning of the experiment. Participants were asked to indicate subjective sleep quality on a 5-point Likert scale (1 = "very bad sleep"; 5 = "very good sleep") and sleep quantity (i.e., the number of hours they slept). In addition, subjective tiredness was measured on a 4-point Likert scale (1 = "not tired at all"; 4 = "very tired").

[2]Trait anxiety (assessed on day 1) and state anxiety (assessed on both days, at the beginning of the experiment) were measured using the German version (Laux et al., 1981) of the State Trait Anxiety Inventory (STAI; Spielberger et al., 1970). The range of possible STAI scores varies from 20 ("minimal") to 80 ("maximal intensity of anxiety").

Laux L, Glanzmann P, Schaffner P, Spielberger CD (1981). Das State-Trait Angstinventar (STAI): Theoretische Grundlagen und Handanweisung [The State-Trait Anxiety Inventory (STAI): theoretical foundations and manual]. Weinheim, Germany: Beltz Test.

Spielberger CD, Gorsuch RL, Lushene RE (1970). STAI manual for the State-Trait Anxiety Inventory. Palo Alto, CA: Consulting Psychologists Press.

B Conditioned and Unconditioned Stimuli

Participants underwent a well-established 2-day fear conditioning/extinction paradigm (Mueller et al., 2014) with acquisition and extinction stages on day 1 and a recall test on day 2. After extinction, participants completed a gambling task (Lueckel et al., 2018) unrelated to the current study.

Conditioned Stimuli (CS). Four different black-and-white male faces (Ekman and Friesen, 1976) with a neutral expression were used as CSs. The faces were assigned to CS types (i.e., CS+E, CS+N, CS-E, CS-N) in a counterbalanced fashion. During each trial, the CS face was presented for 4 s with a size of 13 cm × 18 cm on a black background (22-inch monitor, about 0.80 m from participant), using the computer program Presentation 17.0 (Neurobehavioral Systems, Berkeley, CA, USA). Prior to each trial, a white fixation cross was presented for 1 s. During a jittered intertrial interval (defined as CS offset to CS onset) of 6–8 s, a black screen was shown. As part of a habituation phase, which was performed prior to the acquisition phase, each CS was shown 5 times.

Unconditioned Stimulus (US). We used a 95 dB(A) white noise burst (duration: 1 s) as US, which has previously been shown to elicit a reliable conditioned response for the present paradigm (Sperl et al., 2016). The white noise burst started 3 s after CS onset and was presented by a room speaker. If the 95 dB(A) burst was experienced as too loud, the sound pressure level was reduced to 92 dB(A). Sound pressure level was reduced to 92 dB(A) for one participant each in the yohimbine and sulpiride groups and two participants in the placebo group. The sound pressure level was measured at the participant's head position (approximately 2.30 m from the speaker).

Affective CS Ratings. Participants were asked to rate each CS with regard to its associated arousal (1 = "not arousing"; 5 = "very arousing") and valence (1 = "very pleasant"; 5 = "very unpleasant"), prior to and after each experimental stage. After acquisition, we also assessed the

subjective awareness of the CS–US contingency (0 = “CS was never followed by US”; 3 = “CS was always followed by US”).

C Pharmacological Challenge: Yohimbine, Sulpiride, and Placebo

As explained in the main text, participants received (in a double-blind manner) an oral dose of either 10 mg of yohimbine hydrochloride (HCl), 200 mg of sulpiride, or a placebo pill. Yohimbine (45–75 minutes; Le Verge et al., 1992; Berlan et al., 1993; Grasing et al., 1996; Sturgill et al., 1997; Tam et al., 2001) and sulpiride (3–4 hours; Wiesel et al., 1980; Sugnaux et al., 1983; Mauri et al., 1996) vary in the time they take to reach peak plasma concentrations. To ensure peak plasma levels at a similar time prior to extinction, each participant ingested two capsules (see Figure 1B in the main text). Participants in the sulpiride group received sulpiride 3 hours prior to extinction at t_1 and a placebo pill at t_2. Participants in the yohimbine group received yohimbine 45 minutes prior to extinction at t_2 and a placebo pill at t_1. For participants in the placebo group, both capsules contained placebo pills.

The capsules were compounded by the study pharmacist and were identical in appearance. A cup of water was provided along with the capsules. To control for potential pharmacodynamic or pharmacokinetic food–drug interactions (Koziolek et al., 2019), participants received a standardized breakfast (water and 1–2 bread rolls with jam, hazelnut cocoa spread, cheese, or sausage) between day 1 acquisition and extinction phases. On day 2, participants were asked not to eat for two hours before the experiment. Yohimbine (Ernst and Pittler, 1998) and sulpiride (Rüther et al., 1999) are generally well-tolerated, and adverse side effects are very rare.

Yohimbine. The indole alkaloid yohimbine promotes central and peripheral noradrenaline-release (Goldberg and Robertson, 1983). In the brain, yohimbine acts as antagonist at presynaptic α2-adrenoceptors in the locus coeruleus (Dunlop et al., 2012). Blocking these inhibitory autoreceptors leads to increased locus coeruleus firing and noradrenaline-release (Singewald et al., 2015; Dunlop et al., 2015). To confirm its successful influence on central noradrenaline (Ehlert et al., 2006; Nater and Rohleder, 2009; Ditzen et al., 2014), we assessed salivary α-amylase activity (sAA; see *Supplementary Material, section D*). Beyond the noradrenaline-system, yohimbine also acts on dopamine D2-receptors (Millan et al., 2000; Holmes and Quirk, 2010). Following previous studies (Powers et al., 2009; Meyerbroeker et al., 2012; Smits et al., 2014; Kuehl et al., 2020), we used a single acute dose of 10 mg yohimbine hydrochloride (HCl), which is rapidly absorbed and reaches peak plasma levels within 1 hour (Grasing et al., 1996; Tam et al., 2001). The elimination half-life ranges from 0.25 to 2.5 hours. However, an active yohimbine metabolite (11-hydroxy-yohimbine) shows similar α2-adrenoceptor antagonist properties (Berlan et al., 1993; Tam et al., 2001) and exhibits a longer half-life of around 6 hours (Le Verge et al., 1992; Sturgill et al., 1997). This may explain the relatively long-lasting pharmacodynamic effects.

Sulpiride. The substituted benzamide sulpiride acts as a selective antagonist at pre- and postsynaptic dopamine D2-receptors (Mauri et al., 1996). Sulpiride does not appear to significantly block other receptor types, such as noradrenergic receptors (O'Connor and Brown, 1982; Caley and Weber, 1995). The effects of sulpiride on dopamine depend partly on the dose chosen (Rankin et al., 2010; Crockett and Fehr, 2014; Ford, 2014). High doses (> 400 mg) are thought to exert effects primarily on postsynaptic D2-receptors (Eisenegger et al., 2014; Boschen et al., 2015), thus reducing dopaminergic action (Lai et al., 2013). In contrast, low doses of sulpiride (e.g., 100–200 mg) appear to block mainly presynaptic autoreceptors, which is assumed to result in a net *stimulatory* effect on dopaminergic transmission (Tagliamonte et al., 1975; Kuroki et al., 1999).

Here, we used a single acute dose of 200 mg (Mueller et al., 2011; Chavanon et al., 2013; Ohmann et al., 2020) to *increase* dopamine (Mereu et al., 1983; Kuroki et al., 1999). Sulpiride is only slowly absorbed from the gastrointestinal tract; peak plasma levels occur within 3–4 hours, and the average elimination half-life ranges from 3–10 hours (Wiesel et al., 1980; Sugnaux et al., 1983; Mauri et al., 1996).

Following the recommendations by Crockett and Fehr (2014), we asked participants at the end of day 1 to report their beliefs about whether they had received an active substance (yohimbine or sulpiride) or a placebo pill. The proportion of participants who said that they had received a placebo (yohimbine group: 41%; sulpiride group: 50%; placebo group: 50%) did not differ between groups ($X^2(2) = 0.35$, exact $P = .881$). This indicated successful blinding.

D Salivary α-Amylase

Yohimbine and sulpiride were administered to enhance noradrenergic and dopaminergic transmission, respectively. To confirm the active effect of yohimbine on central noradrenaline release (Ehlert et al., 2006; Nater and Rohleder, 2009; Ditzen et al., 2014), we measured salivary α-amylase activity (sAA). Saliva samples were collected by using the passive drool method on both days at several time points (day 1: 9:30 AM, 11:30 AM, 11:57 AM, 12:07 PM, 12:17 PM, 12:27 PM, 12:37 PM, 1:15 PM, 2:15 PM; day 2: 3:00 PM; see Figure 2 in the main text). Prior to each saliva collection time point, participants were instructed to rinse their mouths with water and to swallow all saliva. Afterward, participants were asked to collect passively the newly produced saliva in their mouths for two minutes and to release the cumulated saliva into a plastic sample tube (SaliCap Set; IBL International, Hamburg, Germany). The specimens were stored at –20 °C until

assay. After thawing for biochemical analysis, samples were centrifuged for 11 minutes at 3,000 rpm, resulting in a clear supernatant. Saliva was diluted 1:400 using 0.9% saline solution. Next, sAA activity was measured using a kinetic colorimetric test and reagents obtained from Roche (Roche Diagnostics, Mannheim, Germany). The intra- and inter-assay coefficients of variance were less than 10%. To correct for skewed distributions, sAA data were $\log_{10}$-transformed. The sAA data of four participants could not be analyzed because the values were below the detection limit (< 3 U/ml; *n* = 1 in the placebo group) or because the values were extremely high (> 800 U/ml; *n* = 1 in the placebo group; *n* = 2 in the sulpiride group).

E Skin Conductance, Electrocardiogram, and Electroencephalogram

Skin conductance, electrocardiogram (ECG), and electroencephalogram (EEG) were recorded at 1,000 Hz using a QuickAmp 72 amplifier (Brain Products, Munich, Germany). The monitor delay (33 ms) was assessed with a Brain Products Photo Sensor, and all marker latencies were corrected accordingly. All physiological data were low-pass filtered online with a cutoff frequency of 200 Hz. Preprocessing was performed in BrainVision Analyzer 2.1.2 (Brain Products, Munich, Germany).

Skin Conductance Responses (SCRs). To assess electrodermal activity (exosomatic measurement, 0.5 V direct current), two Ag/AgCl electrodes of a 10 mm diameter filled with isotonic (0.5% NaCl) electrolyte medium were placed on the thenar/hypothenar sites of the left hand. The raw signal was low-pass filtered (1 Hz, signal amplitude was attenuated by 3 dB at cutoff frequency, 4th order Butterworth filter, 24 dB/octave roll-off) offline in BrainVision Analyzer 2.1.2 (Brain Products, Munich, Germany) and downsampled to 100 Hz. For visual data inspection,

artifact correction, and trough-to-peak analyses, the skin conductance data were exported to Ledalab 3.4.9 (Benedek and Kaernbach, 2010a, 2010b), which was implemented in the MATLAB 9.2 environment (MathWorks, Natick, MA, USA). Technical artifacts were corrected with spline or cubic interpolation. Next, a skin conductance response (SCR) score was calculated for each trial. This was defined as the amplitude-sum of significant SCRs within 1 and 5 s after the CS onset. SCRs during the first second after CS onset were omitted (Boucsein et al., 2012), and SCRs smaller than 0.01 μS were considered to be zero responses. Before averaging, SCR scores were logarithmized, ln(μS+1), to ensure a normal distribution. Afterward, SCR scores for each CS type were averaged across trials. For the acquisition stage, only unreinforced CS+ trials were included to avoid contamination by an evoked response to the US.

Evoked Heart Period (HP). The electrocardiogram (ECG) was measured with pre-gelled Ag/AgCl disc surface electrodes (F-55 type, Megro, Wesel, Germany) in the Lead II configuration (right arm and left leg, ground electrode on left arm). The raw ECG data were band-pass filtered (1−30 Hz, signal amplitude was attenuated by 3 dB at cutoff frequencies, 4th order Butterworth filter, 24 dB/octave roll-off) and notch-filtered (50 ± 2.5 Hz, 16th order Butterworth filter, 96 dB/octave roll-off) offline. Next, R-spikes were detected automatically using the ECG Markers Solution implemented in BrainVision Analyzer 2.1.2 (Brain Products, Munich, Germany). After manual screening, trials with artifacts were rejected and R-spike latencies were corrected if necessary. One participant had to be excluded from ECG analyses for the acquisition stage due to heavy recording artifacts. After artifact correction, a continuous heart period trace was calculated using custom-made MATLAB scripts (MATLAB 9.2; MathWorks, Natick, MA, USA). In particular, we converted the ECG to a time course of interbeat intervals (IBIs). Afterward, each IBI time point reflected the latency between the pre- and succeeding R-spike in ms (Mueller et al., 2013). This IBI time series was then segmented into epochs ranging from –1 to 8 s relative to the

CS onset, baseline-corrected (1 s pre-CS), and averaged across trials for each CS type. Fear conditioning is typically associated with a robust cardiac deceleration for CS+ compared with CS- (Notterman et al., 1952; Deane and Zeaman, 1958; Panitz et al., 2015), which usually overlaps with the US presentation (Deane and Zeaman, 1958; Sperl et al., 2021). Consistent with previous studies (Thigpen et al., 2017; Panitz et al., 2018), the mean heart period change from 2 to 5 s after CS onset was extracted for statistical analyses. For the acquisition stage, only unreinforced CS+ trials were analyzed.

Electroencephalography (EEG). The electroencephalogram (EEG) was recorded with a 64-channel actiCAP active electrode system and actiCAP electrode caps (Brain Products, Munich, Germany), referenced against the average. Raw EEG data were high-pass filtered (0.1 Hz, signal amplitude was attenuated by 3 dB at cutoff frequency, 4th order Butterworth filter, 24 dB/octave roll-off) and notch-filtered (50 ± 2.5 Hz, 16th order Butterworth filter, 96 dB/octave roll-off) offline. Ocular artifacts (eye blinks and movements) were corrected with independent component analysis (extended infomax ICA with classic principal component analysis sphering on the whole artifact-free EEG dataset). To obtain reliable and valid decomposition results (Winkler et al., 2015; Dimigen, 2020), the raw EEG signal was 0.5 Hz high-pass filtered for ICA only. Specifically, ICA weights were trained on the 0.5 Hz high-pass filtered data, ICA matrix files were exported, and, afterward, the "learned" weights ("IC filters") were used to unmix and back-project the 0.1 Hz filtered EEG data (Debener et al., 2010; Winkler et al., 2015). All EEG data were manually screened for artifacts. Intervals that contained artifacts in at least one channel were excluded from further analyses, and corrupted channels were interpolated (spherical spline; Perrin et al., 1989). Finally, EEG was downsampled to 500 Hz. Prior to N170 and LPP analyses, a 30 Hz low-pass filter was applied (signal amplitude was attenuated by 3 dB at cutoff frequency, 4th order Butterworth filter, 24 dB/octave roll-off).

N170 Event-Related Potential (ERP) Component. For analyses of the N170 component, the EEG was referenced against Cz, as this central reference better highlights the N170 at occipito-temporal electrodes (Joyce and Rossion, 2005), which is hypothesized to be generated primarily in the fusiform gyrus (Pizzagalli et al., 2002). Next, event-related potentials (ERPs) were segmented relative to the CS onsets (–200 ms to 400 ms) and baseline-corrected. As expected, the aggregate grand average ERP (Brooks et al., 2017; collapsed across trials of all CS types, across all experimental groups, and across day 1 extinction and day 2 recall) showed a distinct negativity at bilateral occipito-temporal sites during the typical N170 period (Supplementary Figure S2A). Consistent with previous research (Eimer, 2011; Rossion and Jacques, 2012), this negativity was particularly pronounced at T7, TP7, TP9, P7, and PO9 over the left hemisphere, and at T8, TP8, TP10, P8, and PO10 over the right hemisphere. The aggregate grand average pooled across these electrodes showed a negative peak at 165 ms after CS onset. Consequently, we used the mean voltage during the time window from 145 to 185 ms (i.e., the negative peak ± 20 ms) for statistical analyses.

LPP (Late Positive Potential). In the literature, ERPs for LPP analyses are most frequently referenced to the mastoids, which allows emotion-related LPP modulations to be better highlighted (Hajcak et al., 2012). Thus, the EEG was referenced against the average of TP9 and TP10 (mastoids) to analyze LPP responses. Next, we computed ERPs covering 1,000 ms time-locked to the CS onsets. ERPs were baseline-corrected (200 ms pre-stimulus) and averaged across trials of each CS type. The aggregate grand average ERP (Brooks et al., 2017) revealed a sustained positive deflection starting at around 400 ms after CS onset at parieto-occipital electrodes P1, Pz, P2, PO3, POz, PO4, O1, Oz, and O2. A robust positivity was visible from 400 to 800 ms (Supplementary Figure S2B), so we calculated the mean voltage during this time window.

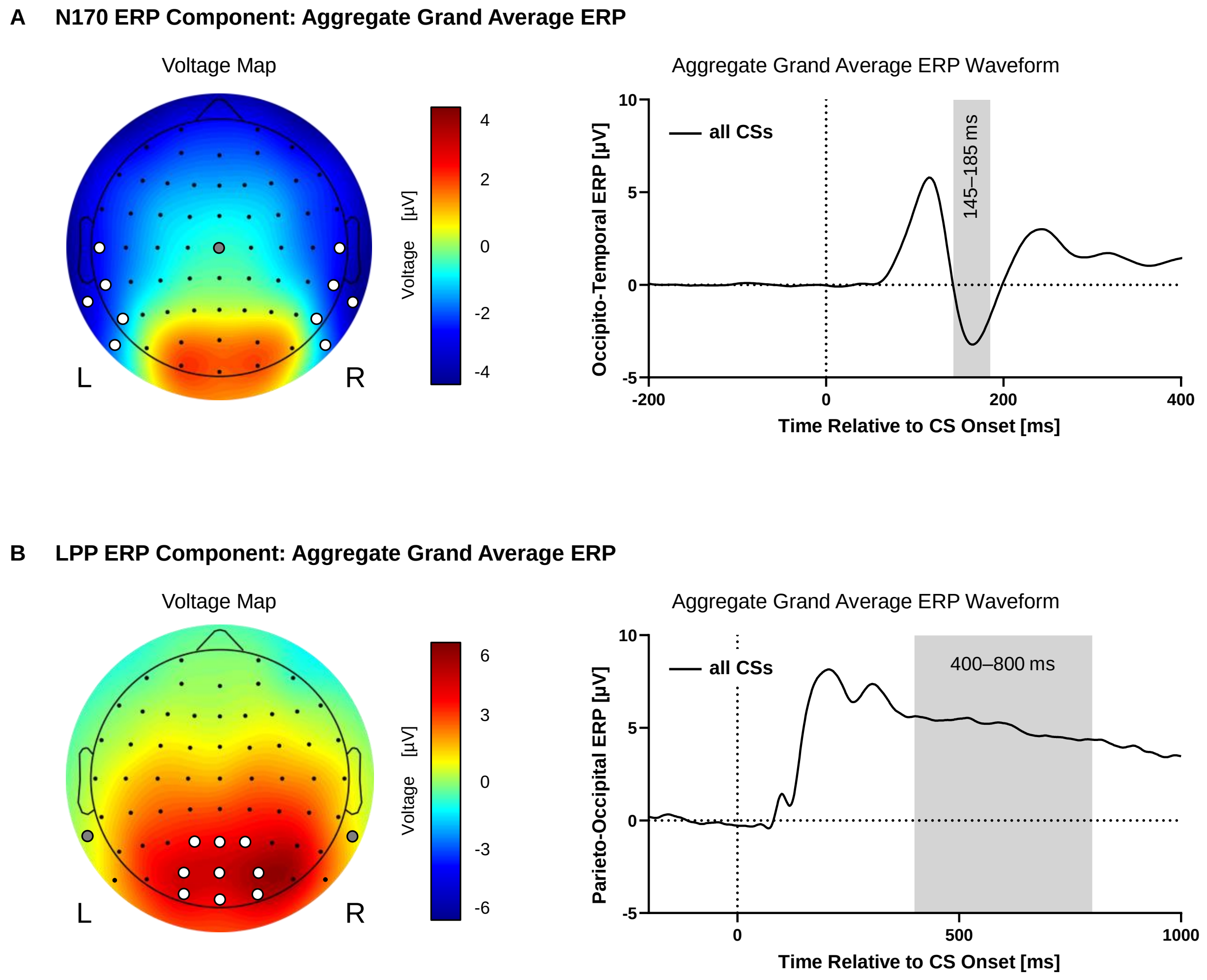

Supplementary Figure S2. The topography (voltage maps, left panels) and waveform (right panels) of the aggregate grand average event-related potential (ERP; collapsed across trials of all CS types, across all experimental groups, and across day 1 extinction and day 2 recall) during the N170 and LPP periods. **(A)** The CSs evoked a distinct negativity at left (T7, TP7, TP9, P7, and PO9) and right (T8, TP8, TP10, P8, and PO10) occipito-temporal electrodes from 145 to 185 ms after CS onset (N170 period). The aforementioned electrodes were included in the ANOVA on N170 amplitudes; they are shown as white dots in the voltage map (left panel). To illustrate the ERP waveform, these electrodes were averaged (right panel). For N170 analyses, EEG data were referenced against Cz (gray dot in voltage map). **(B)** The CSs were associated with a sustained positivity from 400 to 800 ms after CS onset at parieto-occipital electrodes P1, Pz, P2, PO3, POz, PO4, O1, Oz, and O2. These electrodes were included in the ANOVA on LPP amplitudes. They are shown as white dots in the voltage map (left panel) and were averaged to display the ERP waveform (right panel). For LPP analyses, EEG data were referenced against the average of TP9 and TP10 (mastoids, gray dots in voltage map). Gray-shaded areas indicate time windows for statistical analyses. “L” = left hemisphere, “R” = right hemisphere.

F Descriptive Data and Statistical Details

Statistical details for day 1 fear acquisition (Supplementary Table S2), day 1 fear extinction (Supplementary Table S3), and day 2 recall (Supplementary Table S4) are provided on the following pages.

Supplementary Table S2. Day 1 Fear Acquisition: Descriptive (Mean ± Standard Deviation) and Inferential Statistics.

Variable	Yohimbine Group<br>*n* = 17<br>*M* (± *SD*)				Sulpiride Group<br>*n* = 16<br>*M* (± *SD*)				Placebo Group<br>*n* = 18<br>*M* (± *SD*)			
	CS+E	CS-E	CS+N	CS-N	CS+E	CS-E	CS+N	CS-N	CS+E	CS-E	CS+N	CS-N
CS Arousal Ratings[1]	3.24 (± 1.15)	2.12 (± 0.93)	3.06 (± 1.14)	2.06 (± 1.20)	3.06 (± 1.18)	2.31 (± 0.87)	2.69 (± 1.20)	1.94 (± 1.00)	3.44 (± 1.29)	2.33 (± 1.14)	3.28 (± 1.32)	2.28 (± 1.13)
	Inferential Statistical Analysis:<br>ANOVA: *Contingency* (CS+, CS-) × *Later Extinction Status* (E, N) × *Group* (yohimbine, sulpiride, placebo)<br>→ Main effect *Contingency*: $F(1,48) = 27.36$, $P < .001$											
CS Valence Ratings[1]	3.82 (± 1.07)	2.65 (± 1.22)	4.00 (± 0.87)	2.41 (± 1.00)	3.62 (± 1.09)	3.13 (± 1.41)	3.50 (± 0.97)	2.81 (± 1.17)	4.06 (± 0.94)	2.89 (± 1.32)	3.61 (± 1.04)	2.67 (± 0.91)
	Inferential Statistical Analysis:<br>ANOVA: *Contingency* (CS+, CS-) × *Later Extinction Status* (E, N) × *Group* (yohimbine, sulpiride, placebo)<br>→ Main effect *Contingency*: $F(1,48) = 23.46$, $P < .001$											
CS-evoked SCRs[2]	0.07 (± 0.05)	0.05 (± 0.03)	0.08 (± 0.06)	0.06 (± 0.04)	0.04 (± 0.06)	0.03 (± 0.03)	0.05 (± 0.05)	0.03 (± 0.02)	0.07 (± 0.07)	0.04 (± 0.04)	0.06 (± 0.05)	0.04 (± 0.04)
	Inferential Statistical Analysis:<br>ANOVA: *Contingency* (CS+, CS-) × *Later Extinction Status* (E, N) × *Group* (yohimbine, sulpiride, placebo)<br>→ Main effect *Contingency*: $F(1,48) = 15.87$, $P < .001$											

CS-evoked Cardiac Deceleration[3]	34.33 (± 32.90)	13.94 (± 16.86)	27.02 (± 22.83)	9.29 (± 20.24)	20.92 (± 18.14)	10.29 (± 15.81)	18.60 (± 25.44)	2.54 (± 17.51)	34.68 (± 32.09)	18.09 (± 15.26)	34.28 (± 24.85)	8.67 (± 17.56)
	Inferential Statistical Analysis: ANOVA: *Contingency* (CS+, CS-) × *Later Extinction Status* (E, N) × *Group* (yohimbine, sulpiride, placebo) → Main effect *Contingency*: $F(1,47) = 44.94$, $P < .001$											

[1]Participants were asked to rate each CS with regard to its associated arousal (1 = "not arousing"; 5 = "very arousing") and valence (1 = "very pleasant"; 5 = "very unpleasant"). To confirm successful fear conditioning, subjective ratings after the fear acquisition stage were included in the analysis of variance (ANOVA).
[2]CS-evoked skin conductance responses (SCRs) within 1 and 5 s after the CS onset, reported in ln(μS+1), averaged across all acquisition trials, see *Supplementary Material*, *section E*.
[3]CS-evoked cardiac deceleration (measured with electrocardiography, ECG; changes in interbeat intervals in ms) from 2 to 5 s after the CS onset, averaged across all acquisition trials, see *Supplementary Material*, *section E*. One participant in the placebo group had to be excluded from ECG analyses for the acquisition stage due to heavy recording artifacts.

Supplementary Table S3. Day 1 Fear Extinction: Descriptive (Mean ± Standard Deviation) and Inferential Statistics.

Variable	Yohimbine Group *n* = 17 *M* (± *SD*)				Sulpiride Group *n* = 16 *M* (± *SD*)				Placebo Group *n* = 18 *M* (± *SD*)			
	CS+E	CS-E	CS+N	CS-N	CS+E	CS-E	CS+N	CS-N	CS+E	CS-E	CS+N	CS-N
CS Arousal Ratings[1]	Before Extinction:											
	2.53 (± 0.94)	2.24 (± 0.83)	---	---	2.94 (± 1.00)	2.31 (± 0.70)	---	---	2.83 (± 1.20)	2.00 (± 0.97)	---	---
	After Extinction:											
	2.65 (± 1.06)	2.12 (± 0.78)	---	---	2.69 (± 1.08)	2.06 (± 0.77)	---	---	2.89 (± 1.28)	2.33 (± 1.03)	---	---
	Inferential Statistical Analysis: ANOVA: *Contingency* (CS+, CS-) × *Time* (before/after extinction) × *Group* (yohimbine, sulpiride, placebo) → Main effect *Contingency*: $F(1,48) = 20.89$, $P < .001$											

CS Valence Ratings[1]	Before Extinction:											
	3.06 (± 0.66)	3.00 (± 0.94)	---	---	3.38 (± 0.89)	3.06 (± 1.24)	---	---	3.39 (± 1.15)	3.11 (± 0.96)	---	---
	After Extinction:											
	3.18 (± 0.95)	2.76 (± 0.90)	---	---	2.94 (± 0.93)	2.94 (± 1.24)	---	---	3.06 (± 1.21)	2.89 (± 1.08)	---	---
	Inferential Statistical Analysis: ANOVA: *Contingency* (CS+, CS-) × *Time* (before/after extinction) × *Group* (yohimbine, sulpiride, placebo) → No significant main effects or interactions involving *Contingency* (all *P*s ≥ .081)											
CS-evoked SCRs[2]	First 10 Extinction Trials:											
	0.18 (± 0.14)	0.15 (± 0.19)	---	---	0.12 (± 0.08)	0.11 (± 0.11)	---	---	0.17 (± 0.15)	0.13 (± 0.12)	---	---
	Last 10 Extinction Trials:											
	0.06 (± 0.06)	0.06 (± 0.06)	---	---	0.06 (± 0.07)	0.08 (± 0.08)	---	---	0.09 (± 0.13)	0.06 (± 0.07)	---	---
	Inferential Statistical Analysis: ANOVA: *Contingency* (CS+, CS-) × *Time* (first/last 10 extinction trials) × *Group* (yohimbine, sulpiride, placebo) → Main effect *Contingency*: $F(1,48) = 4.09$, $P = .049$											
CS-evoked Cardiac Deceleration[3]	First 10 Extinction Trials:											
	24.60 (± 31.89)	27.18 (± 35.63)	---	---	19.41 (± 33.85)	20.73 (± 23.21)	---	---	40.88 (± 35.06)	34.79 (± 39.23)	---	---
	Last 10 Extinction Trials:											
	2.02 (± 44.33)	11.09 (± 40.97)	---	---	-2.11 (± 31.15)	-7.05 (± 23.07)	---	---	20.74 (± 26.09)	24.90 (± 32.00)	---	---
	Inferential Statistical Analysis: ANOVA: *Contingency* (CS+, CS-) × *Time* (first/last 10 extinction trials) × *Group* (yohimbine, sulpiride, placebo) → No significant main effects or interactions involving *Contingency* (all *P*s ≥ .569)											
EEG N170 Amplitude (145–185 ms)[4]	Electrode T7:											
	-2.82 (± 2.69)	-3.51 (± 3.18)	---	---	-2.84 (± 2.29)	-3.27 (± 2.29)	---	---	-2.88 (± 3.07)	-3.76 (± 3.71)	---	---

Electrode TP7:											
-2.70	-3.45	---	---	-2.62	-3.03	---	---	-3.62	-3.53	---	---
(± 2.65)	(± 3.22)			(± 3.04)	(± 3.08)			(± 3.57)	(± 4.40)		
Electrode TP9:											
-3.73	-4.27	---	---	-4.31	-4.29	---	---	-4.55	-4.58	---	---
(± 3.15)	(± 3.48)			(± 3.35)	(± 3.32)			(± 4.75)	(± 5.32)		
Electrode P7:											
-1.54	-2.21	---	---	-1.65	-1.91	---	---	-2.66	-3.15	---	---
(± 3.19)	(± 4.22)			(± 4.21)	(± 4.63)			(± 4.89)	(± 5.15)		
Electrode PO9:											
-1.10	-1.39	---	---	-1.29	-2.11	---	---	-1.02	-1.08	---	---
(± 4.04)	(± 4.97)			(± 4.86)	(± 5.68)			(± 4.57)	(± 5.22)		
Electrode T8:											
-3.17	-3.17	---	---	-3.56	-4.18	---	---	-3.27	-3.23	---	---
(± 2.98)	(± 2.91)			(± 2.67)	(± 2.69)			(± 3.53)	(± 3.93)		
Electrode TP8:											
-3.21	-3.23	---	---	-3.62	-3.95	---	---	-3.41	-3.46	---	---
(± 2.88)	(± 3.20)			(± 3.77)	(± 3.46)			(± 4.24)	(± 4.33)		
Electrode TP10:											
-4.19	-4.35	---	---	-5.59	-5.35	---	---	-5.19	-4.99	---	---
(± 3.11)	(± 3.79)			(± 4.39)	(± 4.13)			(± 5.49)	(± 5.36)		
Electrode P8:											
-1.59	-1.77	---	---	-1.94	-2.24	---	---	-2.17	-2.53	---	---
(± 3.55)	(± 3.76)			(± 4.53)	(± 4.33)			(± 4.63)	(± 4.95)		
Electrode PO10:											
-1.10	-1.24	---	---	-1.80	-2.32	---	---	-0.86	-0.69	---	---
(± 3.63)	(± 4.37)			(± 4.40)	(± 4.53)			(± 4.88)	(± 4.85)		

Inferential Statistical Analysis:

ANOVA: *Contingency* (CS+, CS-) × *Hemisphere* (left, right) × *Electrode* (T7/8, TP7/8, TP9/10, P7/8, PO9/10) × *Group* (yohimbine, sulpiride, placebo)

→ No significant main effects or interactions involving *Contingency* (all *P*s ≥ .179)

EEG LPP Amplitude (400–800 ms)[5]	Electrode P1:											
	6.50<br>(± 4.27)	5.42<br>(± 3.37)	---	---	5.44<br>(± 3.42)	4.15<br>(± 3.46)	---	---	5.09<br>(± 2.60)	4.49<br>(± 2.13)	---	---
	Electrode Pz:											
	6.12<br>(± 4.17)	5.98<br>(± 3.16)	---	---	5.46<br>(± 3.91)	4.43<br>(± 3.34)	---	---	5.21<br>(± 2.76)	4.74<br>(± 3.73)	---	---
	Electrode P2:											
	6.68<br>(± 4.27)	6.18<br>(± 3.05)	---	---	5.98<br>(± 3.58)	4.99<br>(± 3.77)	---	---	4.86<br>(± 3.17)	5.02<br>(± 2.68)	---	---
	Electrode PO3:											
	6.55<br>(± 4.12)	5.49<br>(± 3.71)	---	---	5.51<br>(± 2.77)	4.61<br>(± 3.35)	---	---	5.66<br>(± 2.33)	5.12<br>(± 2.83)	---	---
	Electrode POz:											
	6.57<br>(± 4.86)	6.20<br>(± 4.01)	---	---	5.52<br>(± 3.13)	4.71<br>(± 3.47)	---	---	5.99<br>(± 2.61)	5.47<br>(± 3.01)	---	---
	Electrode PO4:											
	7.51<br>(± 4.64)	6.20<br>(± 3.42)	---	---	6.32<br>(± 3.31)	5.38<br>(± 3.12)	---	---	6.31<br>(± 3.30)	5.94<br>(± 3.03)	---	---
	Electrode O1:											
	5.05<br>(± 3.23)	4.92<br>(± 3.07)	---	---	5.14<br>(± 2.31)	4.14<br>(± 3.27)	---	---	5.50<br>(± 3.13)	5.24<br>(± 3.69)	---	---
	Electrode Oz:											
	5.22<br>(± 4.11)	4.00<br>(± 3.04)	---	---	4.88<br>(± 2.44)	3.83<br>(± 2.95)	---	---	4.97<br>(± 3.22)	5.16<br>(± 3.45)	---	---
	Electrode O2:											
	6.35<br>(± 4.14)	5.12<br>(± 3.40)	---	---	5.58<br>(± 2.33)	5.16<br>(± 2.86)	---	---	5.92<br>(± 3.10)	6.08<br>(± 3.75)	---	---
	<u>Inferential Statistical Analysis:</u><br>ANOVA: *Contingency* (CS+, CS-) × *Electrode* (P1, Pz, P2, PO3, POz, PO4, O1, Oz, O2) × *Group* (yohimbine, sulpiride, placebo)<br>→ No significant main effects or interactions involving *Contingency* (all *P*s ≥ .083)											

[1]Participants were asked to rate each CS with regard to its associated arousal (1 = "not arousing"; 5 = "very arousing") and valence (1 = "very pleasant"; 5 = "very unpleasant"). To assess fear extinction, subjective ratings before and after the fear extinction stage were included in the analysis of variance (ANOVA). Note that non-extinguished stimuli (CS+N and CS-N) were *not* shown (and *not* rated) during extinction training.
[2]CS-evoked skin conductance responses (SCRs) within 1 and 5 s after the CS onset, reported in ln(μS+1), see *Supplementary Material, section E*. To assess fear extinction, SCRs during the first and last 10 extinction training trials were included in the analysis of variance (ANOVA).
[3]CS-evoked cardiac deceleration (measured with electrocardiography, ECG; changes in interbeat intervals in ms) from 2 to 5 s after the CS onset, see *Supplementary Material, section E*. To assess fear extinction, heart period (i.e., interbeat interval) changes during the first and last 10 extinction training trials were included in the analysis of variance (ANOVA).
[4]CS-evoked N170 event-related potential (ERP) component (measured with electroencephalography, EEG), mean voltage changes (in μV) during the time window from 145 to 185 ms at bilateral occipito-temporal electrodes (T7, TP7, TP9, P7, and PO9 over the left hemisphere, and T8, TP8, TP10, P8, and PO10 over the right hemisphere), see *Supplementary Material, section E*. To achieve a sufficient signal-to-noise ratio for EEG analyses (Huffmeijer et al., 2014), all extinction training trials were averaged.
[5]CS-evoked late positive potential (LPP), which reflects a late-latency event-related potential (ERP) component (measured with electroencephalography, EEG), mean voltage changes (in μV) during the time window from 400 to 800 ms at parieto-occipital electrodes (P1, Pz, P2, PO3, POz, PO4, O1, Oz, and O2), see *Supplementary Material, section E*. To achieve a sufficient signal-to-noise ratio for EEG analyses (Huffmeijer et al., 2014), all extinction training trials were averaged.

Supplementary Table S4. Day 2 Recall Test: Descriptive (Mean ± Standard Deviation) and Inferential Statistics.

Variable	Yohimbine Group *n* = 17 *M* (± *SD*)				Sulpiride Group *n* = 16 *M* (± *SD*)				Placebo Group *n* = 18 *M* (± *SD*)			
	CS+E	CS-E	CS+N	CS-N	CS+E	CS-E	CS+N	CS-N	CS+E	CS-E	CS+N	CS-N
CS Arousal Ratings[1]	2.76 (± 1.15)	2.00 (± 0.87)	2.53 (± 1.01)	2.06 (± 1.09)	2.94 (± 1.12)	2.12 (± 0.72)	2.44 (± 0.89)	2.00 (± 0.89)	2.67 (± 1.14)	2.28 (± 0.96)	2.83 (± 1.20)	2.22 (± 0.94)
	Inferential Statistical Analysis: ANOVA: *Contingency* (CS+, CS-) × *Extinction Status* (E, N) × *Group* (yohimbine, sulpiride, placebo) → Main effect *Contingency*: $F(1,48) = 25.74$, $P < .001$											

CS Valence Ratings[1]	3.24 (± 1.03)	3.06 (± 1.03)	3.59 (± 0.80)	2.94 (± 0.90)	3.37 (± 1.03)	3.38 (± 1.15)	3.31 (± 1.01)	3.12 (± 1.09)	3.33 (± 1.14)	3.11 (± 1.02)	3.22 (± 1.31)	2.94 (± 0.80)
	<u>Inferential Statistical Analysis:</u><br>ANOVA: *Contingency* (CS+, CS-) × *Extinction Status* (E, N) × *Group* (yohimbine, sulpiride, placebo)<br>→ No significant main effects or interactions involving *Contingency* (all *P*s ≥ .159)											
CS-evoked SCRs[2]	0.17 (± 0.15)	0.11 (± 0.10)	0.13 (± 0.09)	0.11 (± 0.09)	0.17 (± 0.17)	0.12 (± 0.12)	0.13 (± 0.12)	0.11 (± 0.13)	0.12 (± 0.15)	0.08 (± 0.07)	0.11 (± 0.14)	0.05 (± 0.05)
	<u>Inferential Statistical Analysis:</u><br>ANOVA: *Contingency* (CS+, CS-) × *Extinction Status* (E, N) × *Group* (yohimbine, sulpiride, placebo)<br>→ Main effect *Contingency*: $F(1,48) = 8.79$, $P = .005$											
CS-evoked Cardiac Deceleration[3]	11.58 (± 49.68)	13.76 (± 30.64)	26.85 (± 37.39)	-5.56 (± 46.30)	15.82 (± 43.22)	4.45 (± 29.43)	12.93 (± 29.34)	11.25 (± 28.08)	24.55 (± 30.50)	16.95 (± 21.15)	13.74 (± 32.29)	19.76 (± 22.37)
	<u>Inferential Statistical Analysis:</u><br>ANOVA: *Contingency* (CS+, CS-) × *Extinction Status* (E, N) × *Group* (yohimbine, sulpiride, placebo)<br>→ Interaction *Contingency* × *Extinction Status* × *Group*: $F(2,48) = 4.27$, $P = .020$<br>Follow-up ANOVAs within the three groups: *Contingency* (CS+, CS-) × *Extinction Status* (E, N)<br>→ Yohimbine Group: Interaction *Contingency* × *Extinction Status*: $F(1,16) = 4.70$, $P = .046$<br>CS+E versus CS-E: $t(16) = -0.17$, $P = .870$<br>CS+N versus CS-N: $t(16) = 2.68$, $P = .016$<br>→ Sulpiride Group: No significant main effects or interactions involving *Contingency* (all *P*s ≥ .370)<br>→ Placebo Group: No significant main effects or interactions involving *Contingency* (all *P*s ≥ .261)											
EEG N170 Amplitude (145–185 ms)[4]	Electrode T7:											
	-2.88 (± 1.54)	-3.01 (± 2.20)	-3.33 (± 2.34)	-2.51 (± 1.92)	-2.25 (± 2.42)	-2.78 (± 1.90)	-2.72 (± 1.90)	-2.63 (± 1.68)	-3.27 (± 4.45)	-3.49 (± 3.84)	-3.30 (± 3.38)	-3.59 (± 4.79)
	Electrode TP7:											
	-2.32 (± 2.05)	-2.63 (± 1.77)	-2.96 (± 2.10)	-2.03 (± 2.05)	-1.64 (± 3.06)	-2.26 (± 1.94)	-2.40 (± 2.46)	-2.05 (± 2.18)	-2.91 (± 4.31)	-3.54 (± 4.31)	-2.99 (± 3.68)	-3.13 (± 4.52)
	Electrode TP9:											
	-3.26 (± 2.70)	-3.30 (± 2.78)	-3.95 (± 2.71)	-2.72 (± 2.61)	-2.99 (± 3.32)	-3.71 (± 2.40)	-3.50 (± 2.36)	-3.50 (± 2.73)	-3.97 (± 5.95)	-4.28 (± 5.41)	-3.97 (± 4.81)	-4.33 (± 5.68)
	Electrode P7:											
	-1.16	-0.97	-1.28	-0.78	-0.46	-1.36	-1.01	-0.72	-1.76	-1.89	-2.11	-1.99

(± 2.86)	(± 2.45)	(± 1.93)	(± 2.22)	(± 4.04)	(± 3.43)	(± 3.71)	(± 3.73)	(± 5.27)	(± 5.01)	(± 4.90)	(± 5.31)
Electrode PO9:											
-0.06	0.22	-0.48	0.05	0.02	-1.10	-0.31	-0.32	0.89	-0.14	0.32	0.45
(± 4.56)	(± 3.58)	(± 2.95)	(± 3.70)	(± 5.25)	(± 4.40)	(± 4.61)	(± 4.93)	(± 5.63)	(± 5.79)	(± 5.69)	(± 5.79)
Electrode T8:											
-3.00	-2.84	-3.05	-2.66	-2.60	-3.03	-2.98	-2.96	-2.93	-2.86	-2.68	-2.86
(± 2.44)	(± 2.70)	(± 2.31)	(± 2.04)	(± 2.78)	(± 2.07)	(± 1.97)	(± 2.27)	(± 3.29)	(± 3.43)	(± 3.04)	(± 3.83)
Electrode TP8:											
-2.81	-2.48	-3.44	-2.46	-2.38	-2.97	-2.43	-2.65	-2.92	-2.88	-2.88	-3.09
(± 2.56)	(± 2.40)	(± 2.12)	(± 1.99)	(± 3.04)	(± 2.43)	(± 2.64)	(± 2.84)	(± 4.10)	(± 4.36)	(± 3.98)	(± 5.53)
Electrode TP10:											
-3.40	-3.62	-4.23	-3.02	-3.61	-4.25	-3.80	-4.37	-3.78	-4.21	-3.57	-4.24
(± 3.15)	(± 2.60)	(± 2.75)	(± 2.35)	(± 3.61)	(± 3.47)	(± 3.44)	(± 3.47)	(± 5.09)	(± 4.85)	(± 4.63)	(± 5.40)
Electrode P8:											
-0.96	-1.22	-2.11	-0.78	0.01	-0.76	0.08	-0.87	-0.86	-0.65	-1.12	-1.10
(± 3.73)	(± 3.45)	(± 2.54)	(± 3.07)	(± 4.08)	(± 3.74)	(± 4.01)	(± 4.43)	(± 4.63)	(± 4.53)	(± 4.71)	(± 5.80)
Electrode PO10:											
-0.49	-0.40	-1.34	-0.13	0.34	-0.56	0.12	-0.44	0.94	0.46	0.38	0.68
(± 3.45)	(± 3.20)	(± 2.78)	(± 3.32)	(± 4.87)	(± 3.66)	(± 4.27)	(± 4.27)	(± 5.44)	(± 5.24)	(± 5.74)	(± 6.03)

Inferential Statistical Analysis:

ANOVA: *Contingency* (CS+, CS-) × *Extinction Status* (E, N) × *Hemisphere* (left, right) × *Electrode* (T7/8, TP7/8, TP9/10, P7/8, PO9/10) × *Group* (yohimbine, sulpiride, placebo)

→ Interaction *Contingency* × *Extinction Status* × *Hemisphere* × *Electrode* × *Group*: $F(8,192) = 2.60$, $P = .016$

Follow-up ANOVAs within the three groups: *Contingency* (CS+, CS-) × *Extinction Status* (E, N) × *Hemisphere* (left, right) × *Electrode* (T7/8, TP7/8, TP9/10, P7/8, PO9/10)

→ Yohimbine Group: Interaction *Contingency* × *Extinction Status* × *Hemisphere* × *Electrode*: $F(4,64) = 5.30$, $P < .001$

Electrode TP10 (right brain hemisphere): Interaction *Contingency* × *Extinction Status*: $F(1,16) = 7.72$, $P = .013$

CS+E versus CS-E: $t(16) = 0.66$, $P = .517$

CS+N versus CS-N: $t(16) = -2.34$, $P = .033$

Electrode P8 (right brain hemisphere): Interaction *Contingency* × *Extinction Status*: $F(1,16) = 10.26$, $P = .006$

CS+E versus CS-E: $t(16) = 0.70$, $P = .496$

CS+N versus CS-N: $t(16) = -3.03$, $P = .008$

Electrode PO10 (right brain hemisphere): Interaction *Contingency* × *Extinction Status*: $F(1,16) = 4.99$, $P = .040$

CS+E versus CS-E: $t(16) = -0.29$, $P = .774$
CS+N versus CS-N: $t(16) = -2.59$, $P = .020$
→ Sulpiride Group: No significant main effects or interactions involving *Contingency* (all *P*s ≥ .099)
→ Placebo Group: No significant main effects or interactions involving *Contingency* (all *P*s ≥ .093)

EEG LPP Amplitude (400–800 ms)[5]	Electrode P1:											
	4.52 (± 4.92)	3.74 (± 3.48)	5.51 (± 4.38)	3.73 (± 3.82)	3.15 (± 3.03)	2.96 (± 4.17)	3.00 (± 2.02)	3.17 (± 3.41)	4.73 (± 2.45)	4.15 (± 2.81)	4.32 (± 2.29)	4.94 (± 4.59)
	Electrode Pz:											
	4.95 (± 4.89)	4.06 (± 3.67)	5.59 (± 4.91)	3.80 (± 4.06)	3.34 (± 3.48)	3.39 (± 4.66)	3.44 (± 2.16)	3.33 (± 3.39)	4.31 (± 2.61)	4.03 (± 2.63)	4.28 (± 2.22)	4.48 (± 4.61)
	Electrode P2:											
	4.86 (± 5.01)	4.20 (± 3.49)	5.72 (± 4.92)	3.73 (± 3.75)	3.42 (± 3.75)	3.63 (± 4.34)	3.84 (± 2.43)	3.48 (± 3.52)	4.73 (± 2.82)	4.25 (± 2.88)	4.11 (± 2.34)	4.80 (± 4.91)
	Electrode PO3:											
	4.50 (± 4.95)	4.22 (± 3.39)	5.76 (± 4.79)	3.97 (± 4.08)	4.23 (± 2.98)	3.71 (± 4.39)	4.19 (± 2.92)	3.84 (± 4.10)	6.19 (± 6.14)	5.33 (± 3.65)	5.44 (± 3.98)	6.43 (± 7.01)
	Electrode POz:											
	5.09 (± 5.07)	4.30 (± 4.19)	5.89 (± 5.16)	3.56 (± 4.50)	3.94 (± 3.37)	4.49 (± 3.94)	4.17 (± 2.41)	3.99 (± 3.66)	5.50 (± 3.24)	4.66 (± 2.81)	4.90 (± 2.51)	5.65 (± 4.79)
	Electrode PO4:											
	5.18 (± 4.30)	4.70 (± 3.44)	5.62 (± 4.40)	4.42 (± 4.02)	4.68 (± 3.69)	5.16 (± 4.07)	5.21 (± 2.76)	4.71 (± 3.80)	6.19 (± 3.84)	5.64 (± 2.99)	5.57 (± 2.70)	6.46 (± 4.87)
	Electrode O1:											
	4.33 (± 3.61)	3.89 (± 3.21)	5.21 (± 4.70)	3.36 (± 4.05)	4.68 (± 3.25)	4.68 (± 3.63)	4.69 (± 3.32)	4.26 (± 3.45)	5.60 (± 3.31)	5.14 (± 3.01)	5.13 (± 2.99)	6.02 (± 5.04)
	Electrode Oz:											
	3.48 (± 3.13)	3.31 (± 3.11)	4.73 (± 4.21)	3.04 (± 3.57)	3.85 (± 2.60)	4.07 (± 3.59)	4.61 (± 3.35)	4.10 (± 3.87)	5.41 (± 3.67)	4.78 (± 3.12)	4.73 (± 3.26)	5.74 (± 5.85)
	Electrode O2:											
	3.78 (± 2.94)	3.72 (± 2.39)	4.90 (± 3.25)	3.50 (± 3.18)	4.63 (± 3.36)	4.94 (± 3.67)	5.53 (± 3.11)	4.83 (± 3.65)	6.02 (± 3.53)	5.64 (± 3.39)	5.41 (± 3.12)	6.29 (± 5.38)

<u>Inferential Statistical Analysis:</u>

ANOVA: *Contingency* (CS+, CS-) × *Extinction Status* (E, N) × *Electrode* (P1, Pz, P2, PO3, POz, PO4, O1, Oz, O2) × *Group* (yohimbine, sulpiride, placebo)

→ Interaction *Contingency* × *Extinction Status* × *Group*: $F(2,48) = 3.43$, $P = .041$

Follow-up ANOVAs within the three groups: *Contingency* (CS+, CS-) × *Extinction Status* (E, N) × *Electrode* (P1, Pz, P2, PO3, POz, PO4, O1, Oz, O2)

→ Yohimbine Group: Interaction *Contingency* × *Extinction Status*: $F(1,16) = 4.61$, $P = .047$

CS+E versus CS-E: $t(16) = 1.25$, $P = .229$

CS+N versus CS-N: $t(16) = 3.15$, $P = .006$

→ Sulpiride Group: No significant main effects or interactions involving *Contingency* (all Ps ≥ .256)

→ Placebo Group: No significant main effects or interactions involving *Contingency* (all Ps ≥ .198)

---

[1]Participants were asked to rate each CS with regard to its associated arousal (1 = “not arousing”; 5 = “very arousing”) and valence (1 = “very pleasant”; 5 = “very unpleasant”). To assess fear and extinction recall, subjective ratings before the day 2 recall stage were included in the analysis of variance (ANOVA).

[2]CS-evoked skin conductance responses (SCRs) within 1 and 5 s after the CS onset, reported in ln(µS+1), see *Supplementary Material, section E*. Because of a rapid habituation of fear-conditioned SCRs (Sperl et al., 2019), SCRs during the first 10 recall test trials were included in the analysis of variance (ANOVA).

[3]CS-evoked cardiac deceleration (measured with electrocardiography, ECG; changes in interbeat intervals in ms) from 2 to 5 s after the CS onset, see *Supplementary Material, section E*. Because of a rapid habituation of fear-conditioned bradycardia (Panitz et al., 2018), heart period (i.e., interbeat interval) changes during the first 10 recall test trials were included in the analysis of variance (ANOVA).

[4]CS-evoked N170 event-related potential (ERP) component (measured with electroencephalography, EEG), mean voltage changes (in µV) during the time window from 145 to 185 ms at bilateral occipito-temporal electrodes (T7, TP7, TP9, P7, and PO9 over the left hemisphere, and T8, TP8, TP10, P8, and PO10 over the right hemisphere), see *Supplementary Material, section E*. To achieve a sufficient signal-to-noise ratio for EEG analyses (Huffmeijer et al., 2014), all recall test trials were averaged.

[5]CS-evoked late positive potential (LPP), which reflects a late-latency event-related potential (ERP) component (measured with electroencephalography, EEG), mean voltage changes (in µV) during the time window from 400 to 800 ms at parieto-occipital electrodes (P1, Pz, P2, PO3, POz, PO4, O1, Oz, and O2), see *Supplementary Material, section E*. To achieve a sufficient signal-to-noise ratio for EEG analyses (Huffmeijer et al., 2014), all recall test trials were averaged.

G Supplementary Results for Day 1 Fear Acquisition

The key findings of the present study refer to the influence of yohimbine administration (between fear acquisition and extinction) on fear and extinction recall, which was assessed on the following day. For a precise interpretation of our day 2 results, it is a prerequisite to confirm that fear conditioning on day 1 was successful. Therefore, the detailed results for day 1 fear acquisition are provided as *Supplementary Material G* (see also Supplementary Table S2 in *Supplementary Material F* for statistical details).

As expected, affective CS ratings and peripheral physiological responses confirmed successful fear conditioning. Compared with the two CS- (CS-E and CS-N), both CS+ (CS+E and CS+N) were rated as significantly more arousing (see Supplementary Figure S3A) and unpleasant (see Supplementary Figure S3B). The *Contingency* (CS+, CS-) × *Later Extinction Status* (E, N) × *Group* (yohimbine, sulpiride, placebo) ANOVA on CS arousal ratings revealed a significant *Contingency* main effect ($F(1,48) = 27.36$, $P < .001$). Similarly, the ANOVA on CS valence ratings also showed a significant *Contingency* main effect ($F(1,48) = 23.46$, $P < .001$).

On the peripheral physiological level, both CS+ (CS+E and CS+N), relative to the two CS- (CS-E and CS-N), evoked significantly larger SCR amplitudes (see Supplementary Figure S3C) and significantly stronger cardiac deceleration ("fear-conditioned bradycardia"; see Supplementary Figure S4). For SCR data, the *Contingency* (CS+, CS-) × *Later Extinction Status* (E, N) × *Group* (yohimbine, sulpiride, placebo) ANOVA indicated a significant *Contingency* main effect ($F(1,48) = 15.87$, $P < .001$). Finally, a significant *Contingency* main effect ($F(1,47) = 44.94$, $P < .001$) was also observed for heart period data. Given that we found an effect of yohimbine on CS-evoked heart period changes during fear recall on day 2 (see main text), the heart period results during fear

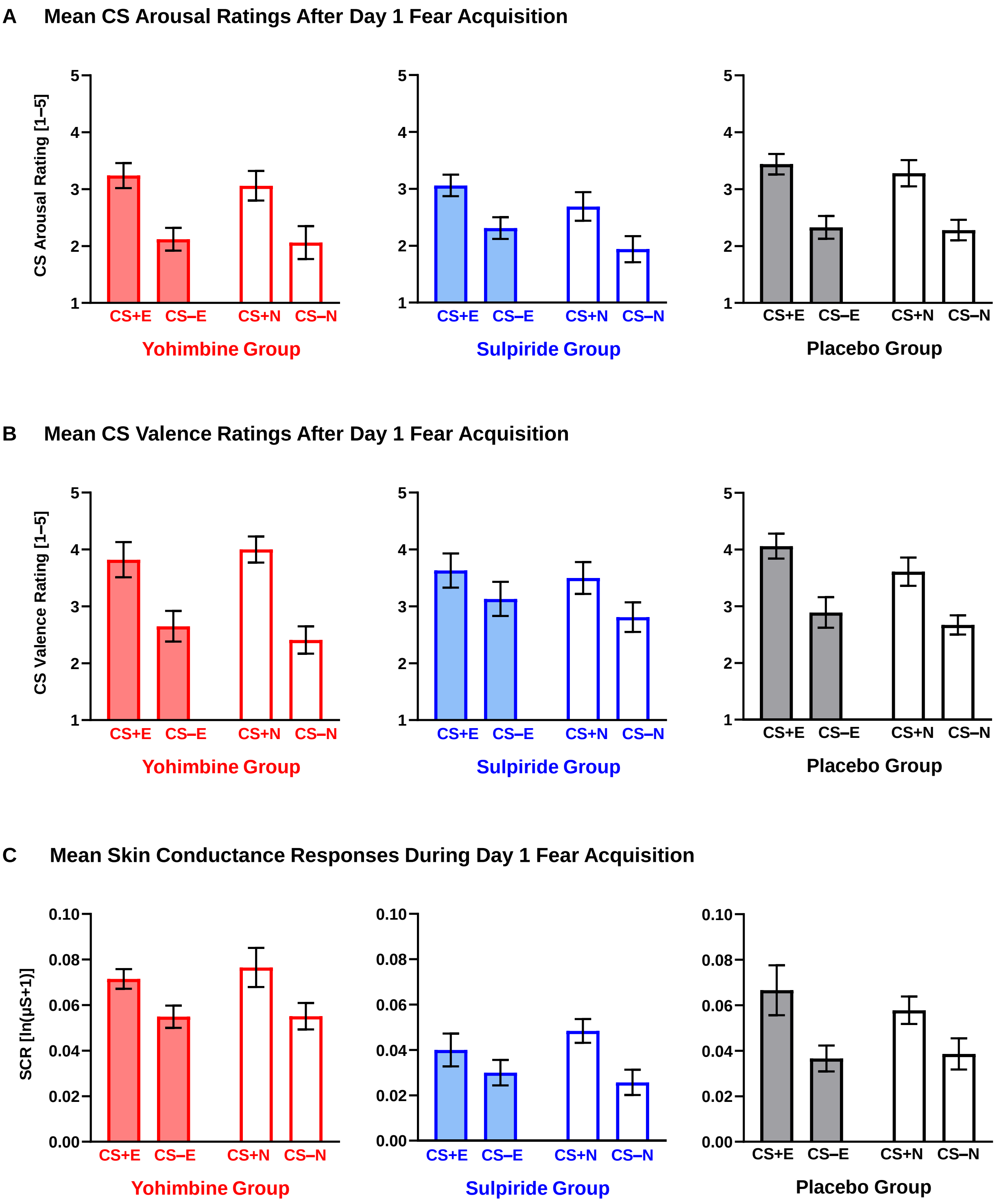

Supplementary Figure S3. (A) Arousal and **(B)** valence ratings of the CSs after fear acquisition as well as **(C)** CS-evoked skin conductance responses (SCRs) during fear acquisition on day 1 confirmed successful fear conditioning. Participants were asked to rate each CS with regard to its associated arousal (1 = "not arousing"; 5 = "very arousing") and valence (1 = "very pleasant"; 5 = "very unpleasant"). Mean (± within-participant *SEM*, adjusted within each group; O'Brien and Cousineau, 2014) arousal/valence ratings and SCR amplitudes for each CS type are displayed.

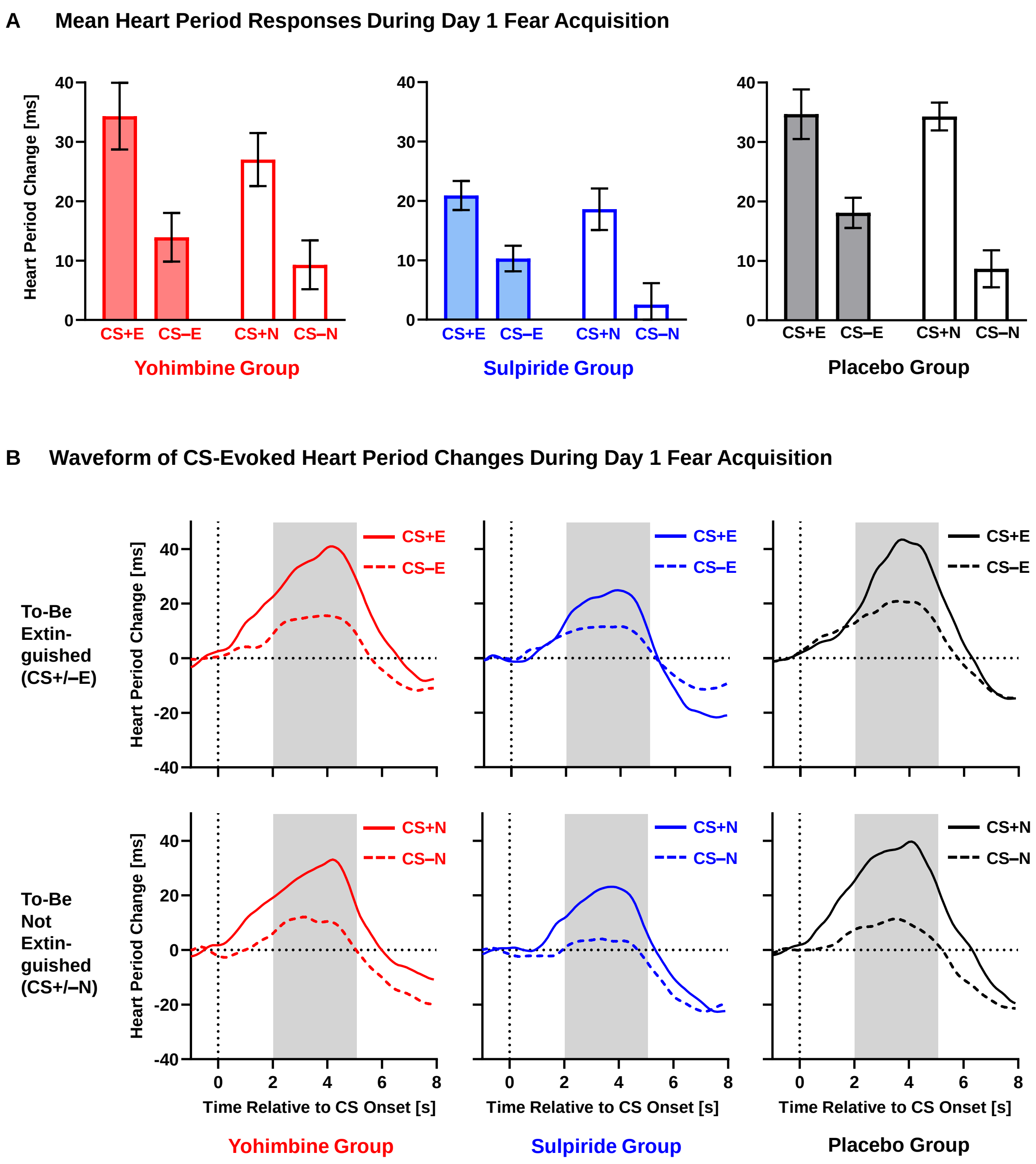

Supplementary Figure S4. (A) CS-evoked heart period changes during fear acquisition on day 1 confirmed successful fear conditioning. Mean (± within-participant *SEM*, adjusted within each group; O'Brien and Cousineau, 2014) heart period changes after CS onset are displayed. **(B)** The waveform of CS-evoked heart period changes is shown for to-be extinguished (CS+E, CS-E; upper panels) and to-be not extinguished (CS+N, CS-N; lower panels) stimuli, separately for the yohimbine (n = 17; left panels), sulpiride (n = 16; middle panels), and placebo groups (n = 18; right panels). Gray-shaded areas indicate time windows for statistical analyses (2–5 s post-CS).

acquisition on day 1 are of particular relevance. Thus, the waveform of CS-evoked heart period changes during fear acquisition on day 1 is displayed in Supplementary Figure S4B. Importantly, fear conditioning was successful for both to-be extinguished and to-be non-extinguished stimuli in each of the three groups. There were no significant interactions including the factors *Later Extinction Status* or *Group* (all Ps $\geq .329$).

References

Benedek M, Kaernbach C (2010a) A continuous measure of phasic electrodermal activity. J Neurosci Methods 190:80–91.

Benedek M, Kaernbach C (2010b) Decomposition of skin conductance data by means of nonnegative deconvolution. Psychophysiology 47:647–658.

Berlan M, Le Verge R, Galitzky J, Le Corre P (1993) α2-adrenoceptor antagonist potencies of two hydroxylated metabolites of yohimbine. Br J Pharmacol 108:927–932.

Bosch JA, Veerman ECI, de Geus EJ, Proctor GB (2011) α-amylase as a reliable and convenient measure of sympathetic activity: don't start salivating just yet! Psychoneuroendocrinology 36:449–453.

Boschen SL, Andreatini R, da Cunha C (2015) Activation of postsynaptic D2 dopamine receptors in the rat dorsolateral striatum prevents the amnestic effect of systemically administered neuroleptics. Behav Brain Res 281:283–289.

Boucsein W, Fowles DC, Grimnes S, Ben-Shakhar G, Roth WT, Dawson ME, Filion DL (2012) Publication recommendations for electrodermal measurements. Psychophysiology 49:1017–1034.

Brooks JL, Zoumpoulaki A, Bowman H (2017) Data-driven region-of-interest selection without inflating type I error rate. Psychophysiology 54:100–113.

Caley CF, Weber SS (1995) Sulpiride: an antipsychotic with selective dopaminergic antagonist properties. Ann Pharmacother 29:152–160.

Chavanon M-L, Wacker J, Stemmler G (2013) Paradoxical dopaminergic drug effects in extraversion: dose- and time-dependent effects of sulpiride on EEG theta activity. Front Hum Neurosci 7:117.

Crockett MJ, Fehr E (2014) Social brains on drugs: tools for neuromodulation in social neuroscience. Soc Cogn Affect Neurosci 9:250–254.

Deane GE, Zeaman D (1958) Human heart rate during anxiety. Percept Mot Skills 8:103–106.

Debener S, Thorne J, Schneider TR, Viola FC (2010) Using ICA for the analysis of multi-channel EEG data. In: Simultaneous EEG and fMRI: recording, analysis, and application (Ullsperger M, Debener S, eds), pp 121–133. New York, NY: Oxford University Press.

Dimigen O (2020) Optimizing the ICA-based removal of ocular EEG artifacts from free viewing experiments. NeuroImage 207:116117.

Ditzen B, Ehlert U, Nater UM (2014) Associations between salivary alpha-amylase and catecholamines—a multilevel modeling approach. Biol Psychol 103:15–18.

Dunlop BW, Mansson E, Gerardi M (2012) Pharmacological innovations for posttraumatic stress disorder and medication-enhanced psychotherapy. Curr Pharm Des 18:5645–5658.

Dunlop BW, Ressler KJ, Rothbaum BO (2015) Pharmacological mechanisms of modulating fear and extinction. In: Primer on anxiety disorders: translational perspectives on diagnosis and treatment (Ressler KJ, Pine DS, Rothbaum BO, eds), pp 367–385. New York, NY: Oxford University Press.

Ehlert U, Erni K, Hebisch G, Nater U (2006) Salivary alpha-amylase levels after yohimbine challenge in healthy men. J Clin Endocrinol Metab 91:5130–5133.

Eimer M (2011) The face-sensitive N170 component of the event-related brain potential. In: The Oxford handbook of face perception (Calder AJ, Rhodes G, Johnson MH, Haxby JV, eds), pp 329–344. New York, NY: Oxford University Press.

Eisenegger C, Naef M, Linssen A, Clark L, Gandamaneni PK, Müller U, Robbins TW (2014) Role of dopamine D2 receptors in human reinforcement learning. Neuropsychopharmacology 39:2366–2375.

Ekman P, Friesen WV (1976) Pictures of facial affect. Palo Alto, CA: Consulting Psychologists Press.

Ernst E, Pittler MH (1998) Yohimbine for erectile dysfunction: a systematic review and meta-analysis of randomized clinical trials. J Urol 159:433–436.

Ford CP (2014) The role of D2-autoreceptors in regulating dopamine neuron activity and transmission. Neuroscience 282:13–22.

Goldberg MR, Robertson D (1983) Yohimbine: a pharmacological probe for study of the alpha 2-adrenoreceptor. Pharmacol Rev 35:143–180.

Grasing K, Sturgill MG, Rosen RC, Trout JR, Thomas TJ, Kulkarni GD, Maines P, Seibold JR (1996) Effects of yohimbine on autonomic measures are determined by individual values for area under the concentration-time curve. J Clin Pharmacol 36:814–822.

Hajcak G, Weinberg A, MacNamara A, Foti D (2012) ERPs and the study of emotion. In: The Oxford handbook of event-related potential components (Luck SJ, Kappenman ES, eds), pp 441–472. New York, NY: Oxford University Press.

Holmes A, Quirk GJ (2010) Pharmacological facilitation of fear extinction and the search for adjunct treatments for anxiety disorders—the case of yohimbine. Trends Pharmacol Sci 31:2–7.

Huffmeijer R, Bakermans-Kranenburg MJ, Alink LRA, van IJzendoorn MH (2014) Reliability of event-related potentials: the influence of number of trials and electrodes. Physiol Behav 130:13–22.

Joyce C, Rossion B (2005) The face-sensitive N170 and VPP components manifest the same brain processes: the effect of reference electrode site. Clin Neurophysiol 116:2613–2631.

Koziolek M et al. (2019) The mechanisms of pharmacokinetic food-drug interactions—a perspective from the UNGAP group. Eur J Pharm Sci 134:31–59.

Kuehl LK, Deuter CE, Hellmann-Regen J, Kaczmarczyk M, Otte C, Wingenfeld K (2020) Enhanced noradrenergic activity by yohimbine and differential fear conditioning in patients with major depression with and without adverse childhood experiences. Prog Neuropsychopharmacol Biol Psychiatry 96:109751.

Kuroki T, Meltzer HY, Ichikawa J (1999) Effects of antipsychotic drugs on extracellular dopamine levels in rat medial prefrontal cortex and nucleus accumbens. J Pharmacol Exp Ther 288:774–781.

Lai EC-C, Chang C-H, Kao Yang Y-H, Lin S-J, Lin C-Y (2013) Effectiveness of sulpiride in adult patients with schizophrenia. Schizophr Bull 39:673–683.

Laux L, Glanzmann P, Schaffner P, Spielberger CD (1981) Das State-Trait Angstinventar (STAI): Theoretische Grundlagen und Handanweisung [The State-Trait Anxiety Inventory (STAI): theoretical foundations and manual]. Weinheim, Germany: Beltz Test.

Le Verge R, Le Corre P, Chevanne F, De Maindreville MD, Royer D, Levy J (1992) Determination of yohimbine and its two hydroxylated metabolites in humans by high-performance liquid chromatography and mass spectral analysis. J Chromatogr B Biomed Sci Appl 574:283–292.

Lueckel M, Panitz C, Nater UM, Mueller EM (2018) Reliability and robustness of feedback-evoked brain-heart coupling after placebo, dopamine, and noradrenaline challenge. Int J Psychophysiol 132:298–310.

Margraf J (1994) Mini-DIPS: Diagnostisches Kurz-Interview bei psychischen Störungen [Diagnostic interview for mental disorders—short version]. Berlin, Germany: Springer.

Mauri MC, Bravin S, Bitetto A, Rudelli R, Invernizzi G (1996) A risk-benefit assessment of sulpiride in the treatment of schizophrenia. Drug Saf 14:288–298.

Mereu G, Casu M, Gessa GL (1983) (—)-Sulpiride activates the firing rate and tyrosine hydroxylase activity of dopaminergic neurons in unanesthetized rats. Brain Res 264:105–110.

Meyerbroeker K, Powers MB, van Stegeren A, Emmelkamp PMG (2012) Does yohimbine hydrochloride facilitate fear extinction in virtual reality treatment of fear of flying? A randomized placebo-controlled trial. Psychother Psychosom 81:29–37.

Millan MJ, Newman-Tancredi A, Audinot V, Cussac D, Lejeune F, Nicolas J-P, Cog F, Galizzi J-P, Boutin JA, Rivet J-M, Dekeyne A, Gobert A (2000) Agonist and antagonist actions of yohimbine as compared to fluparoxan at α2-adrenergic receptors (AR)s, serotonin (5-HT)1A, 5-HT1B, 5-HT1D and dopamine D2 and D3 receptors. Significance for the modulation of frontocortical monoaminergic transmission and depressive states. Synapse 35:79–95.

Mueller EM, Makeig S, Stemmler G, Hennig J, Wacker J (2011) Dopamine effects on human error processing depend on Catechol-O-Methyltransferase VAL158MET genotype. J Neurosci 31:15818–15825.

Mueller EM, Panitz C, Hermann C, Pizzagalli DA (2014) Prefrontal oscillations during recall of conditioned and extinguished fear in humans. J Neurosci 34:7059–7066.

Mueller EM, Stemmler G, Hennig J, Wacker J (2013) 5-HTTLPR and anxiety modulate brain-heart covariation. Psychophysiology 50:441–453.

Nater UM, Rohleder N (2009) Salivary alpha-amylase as a non-invasive biomarker for the sympathetic nervous system: current state of research. Psychoneuroendocrinology 34:486–496.

Notterman JM, Schoenfeld WN, Bersh PJ (1952) Conditioned heart rate response in human beings during experimental anxiety. J Comp Physiol Psychol 45:1–8.

O'Brien F, Cousineau D (2014) Representing error bars in within-subject designs in typical software packages. Quant Method Psychol 10:56–67.

O'Connor SE, Brown RA (1982) The pharmacology of sulpiride—a dopamine receptor antagonist. Gen Pharmacol 13:185–193.

Ohmann HA, Kuper N, Wacker J (2020) A low dosage of the dopamine D2-receptor antagonist sulpiride affects effort allocation for reward regardless of trait extraversion. Personal Neurosci 3:1302.

Panitz C, Hermann C, Mueller EM (2015) Conditioned and extinguished fear modulate functional corticocardiac coupling in humans. Psychophysiology 52:1351–1360.

Panitz C, Sperl MFJ, Hennig J, Klucken T, Hermann C, Mueller EM (2018) Fearfulness, neuroticism/anxiety, and COMT Val158Met in long-term fear conditioning and extinction. Neurobiol Learn Mem 155:7–20.

Perrin F, Pernier J, Bertrand O, Echallier JF (1989) Spherical splines for scalp potential and current density mapping. Electroencephalogr Clin Neurophysiol 72:184–187.

Pizzagalli DA, Lehmann D, Hendrick AM, Regard M, Pascual-Marqui RD, Davidson RJ (2002) Affective judgments of faces modulate early activity (approximately 160 ms) within the fusiform gyri. NeuroImage 16:663–677.

Powers MB, Smits JAJ, Otto MW, Sanders C, Emmelkamp PMG (2009) Facilitation of fear extinction in phobic participants with a novel cognitive enhancer: a randomized placebo controlled trial of yohimbine augmentation. J Anxiety Disord 23:350–356.

Rankin ML, Hazelwood LA, Free RB, Namkung Y, Rex EB, Roof RA, Sibley DR (2010) Molecular pharmacology of the dopamine receptors. In: Dopamine handbook (Iversen LL, Iversen SD, Dunnett SB, Björklund A, eds), pp 63–87. New York, NY: Oxford University Press.

Rossion B, Jacques C (2012) The N170: understanding the time course of face perception in the human brain. In: The Oxford handbook of event-related potential components (Luck SJ, Kappenman ES, eds), pp 115–141. New York, NY: Oxford University Press.

Rüther E, Degner D, Munzel U, Brunner E, Lenhard G, Biehl J, Vögtle-Junkert U (1999) Antidepressant action of sulpiride. Results of a placebo-controlled double-blind trial. Pharmacopsychiatry 32:127–135.

Singewald N, Schmuckermair C, Whittle N, Holmes A, Ressler KJ (2015) Pharmacology of cognitive enhancers for exposure-based therapy of fear, anxiety and trauma-related disorders. Pharmacol Ther 149:150–190.

Smits JAJ, Rosenfield D, Davis ML, Julian K, Handelsman PR, Otto MW, Tuerk P, Shiekh M, Rosenfield B, Hofmann SG, Powers MB (2014) Yohimbine enhancement of exposure therapy for social anxiety disorder: a randomized controlled trial. Biol Psychiatry 75:840–846.

Sperl MFJ, Panitz C, Hermann C, Mueller EM (2016) A pragmatic comparison of noise burst and electric shock unconditioned stimuli for fear conditioning research with many trials. Psychophysiology 53:1352–1365.

Sperl MFJ, Panitz C, Rosso IM, Dillon DG, Kumar P, Hermann A, Whitton AE, Hermann C, Pizzagalli DA, Mueller EM (2019) Fear extinction recall modulates human frontomedial theta and amygdala activity. Cereb Cortex 29:701–715.

Sperl MFJ, Wroblewski A, Mueller M, Straube B, Mueller EM (2021) Learning dynamics of electrophysiological brain signals during human fear conditioning. NeuroImage 226:117569.

Spielberger CD, Gorsuch RL, Lushene RE (1970) STAI manual for the State-Trait Anxiety Inventory. Palo Alto, CA: Consulting Psychologists Press.

Strahler J, Skoluda N, Kappert MB, Nater UM (2017) Simultaneous measurement of salivary cortisol and alpha-amylase: application and recommendations. Neurosci Biobehav Rev 83:657–677.

Sturgill MG, Grasing KW, Rosen RC, Thomas TJ, Kulkarni GD, Trout JR, Maines M, Seibold JR (1997) Yohimbine elimination in normal volunteers is characterized by both one- and two-compartment behavior. J Cardiovasc Pharmacol 29:697–703.

Sugnaux FR, Benakis A, Fonzo D, Di Carlo R (1983) Dose-dependent pharmacokinetics of sulpirideand sulpiride-induced prolactin secretionin man. Eur J Drug Metab Pharmacokinet 8:189–200.

Tagliamonte A, Montis G, Olianas M, Vargiu L, Corsini GU, Gessa GL (1975) Selective increase of brain dopamine synthesis by sulpiride. J Neurochem 24:707–710.

Tam S, Worcel M, Wyllie M (2001) Yohimbine: a clinical review. Pharmacol Ther 91:215–243.

Thigpen NN, Bartsch F, Keil A (2017) The malleability of emotional perception: short-term plasticity in retinotopic neurons accompanies the formation of perceptual biases to threat. J Exp Psychol Gen 146:464–471.

Wiesel FA, Alfredsson G, Ehrnebo M, Sedvall G (1980) The pharmacokinetics of intravenous and oral sulpiride in healthy human subjects. Eur J Clin Pharmacol 17:385–391.

Winkler I, Debener S, Müller K-R, Tangermann M (2015) On the influence of high-pass filtering on ICA-based artifact reduction in EEG-ERP. Annu Int Conf IEEE Eng Med Biol Soc 37:4101–4105.
